# Representing Organic Matter Thermodynamics in Biogeochemical Reactions via Substrate-Explicit Modeling

**DOI:** 10.3389/fmicb.2020.531756

**Published:** 2020-10-23

**Authors:** Hyun-Seob Song, James C. Stegen, Emily B. Graham, Joon-Yong Lee, Vanessa A. Garayburu-Caruso, William C. Nelson, Xingyuan Chen, J. David Moulton, Timothy D. Scheibe

**Affiliations:** ^1^Pacific Northwest National Laboratory, Richland, WA, United States; ^2^Departments of Biological Systems Engineering and Food Science and Technology, University of Nebraska-Lincoln, Lincoln, NE, United States; ^3^Los Alamos National Laboratory, Los Alamos, NM, United States

**Keywords:** substrate-explicit modeling, microbial-explicit modeling, enzyme-explicit modeling, integrative biogeochemical modeling, high-resolution metabolomics, FTICR-MS

## Abstract

Predictive biogeochemical modeling requires data-model integration that enables explicit representation of the sophisticated roles of microbial processes that transform substrates. Data from high-resolution organic matter (OM) characterization are increasingly available and can serve as a critical resource for this purpose, but their incorporation into biogeochemical models is often prohibited due to an over-simplified description of reaction networks. To fill this gap, we proposed a new concept of biogeochemical modeling—termed *substrate-explicit modeling*—that enables parameterizing OM-specific oxidative degradation pathways and reaction rates based on the thermodynamic properties of OM pools. Based on previous developments in the literature, we characterized the resulting kinetic models by only two parameters regardless of the complexity of OM profiles, which can greatly facilitate the integration with reactive transport models for ecosystem simulations by alleviating the difficulty in parameter identification. The two parameters include maximal growth rate (μ^max^) and harvest volume (V_h_) (i.e., the volume that a microbe can access for harvesting energy). For every detected organic molecule in a given sample, our approach provides a systematic way to formulate reaction kinetics from chemical formula, which enables the evaluation of the impact of OM character on biogeochemical processes across conditions. In a case study of two sites with distinct OM thermodynamics using ultra high-resolution metabolomics datasets derived from Fourier transform ion cyclotron resonance mass spectrometry analyses, our method predicted how oxidative degradation is primarily driven by thermodynamic efficiency of OM consistent with experimental rate measurements (as shown by correlation coefficients of up to 0.61), and how biogeochemical reactions can vary in response to carbon and/or oxygen limitations. Lastly, we showed that incorporation of enzymatic regulations into substrate-explicit models is critical for more reasonable predictions. This result led us to present *integrative biogeochemical modeling* as a unifying framework that can ideally describe the dynamic interplay among microbes, enzymes, and substrates to address advanced questions and hypotheses in future studies. Altogether, the new modeling concept we propose in this work provides a foundational platform for unprecedented predictions of biogeochemical and ecosystem dynamics through enhanced integration with diverse experimental data and extant modeling approaches.

## Introduction

Organic matter (OM) is a key determinant of global biogeochemistry and exerts far-reaching impacts on regional and global ecosystem health. Degradation of complex OM is primarily driven by microbial and enzymatic activities around environmental substrates (Moorhead et al., 2013; Manzoni et al., 2016; Paul, 2016; Varjani, 2017). Therefore, a proper representation of the dynamic interplay among microbes, enzymes, and substrates is key for reliable prediction of OM cycling and ecosystem functioning. Despite an increasing ability to generate high-resolution data for each of these components, understanding of fundamental processes that govern their interplay is limited and, consequently, is poorly represented in models (Fatichi et al., 2019). To date, no current-generation modeling framework has been available to interpret and utilize increasingly available high-resolution metabolite data for predicting biogeochemical cycles at the ecosystem level.

Earlier biogeochemical models provide a lumped description of microbial and enzymatic activities, as well as chemical entities (Schimel and Schaeffer, 2012; Blankinship et al., 2018). This reductionist approach significantly limits the predictive ability of models. For the past decade, growing recognition of the significance of microbial activities on biogeochemistry has led to the development of microbial-explicit models (MXMs) (Todd-Brown et al., 2012; Wieder et al., 2015; Allison, 2017; Sulman et al., 2018), which include DEMENT (Allison, 2012), CORPSE (Sulman et al., 2014), MIMICS (Wieder et al., 2014), MEND (Wang et al., 2015), RESOM (Tang and Riley, 2015), and other functional guild-based models (Hood et al., 2006; Jin and Roden, 2011; Bouskill et al., 2012). With or without coupled consideration of enzymatic processes, these models account for microbial physiology and interactions to provide a deeper understanding of microbially mediated OM decomposition.

Enzyme-based or enzyme-explicit models (EXMs) are a complementary approach that collectively describes biogeochemical functions performed by a microbial community with a focus on extracellular enzymes, alleviating the difficulty in identifying functional traits of individual organisms or their groups (Moorhead et al., 2013; Song et al., 2014, 2017; Song and Liu, 2015). In contrast with such increasing details considered in MXM and EXM, over-simplified descriptions of substrate pools remain as a serious *bottleneck* in building predictive biogeochemical models because further elaboration of microbial and enzyme activities becomes difficult without expanded consideration of complex OM chemistry that influences the specific metabolic pathways employed by microorganisms.

Extension of biogeochemical models to include detailed OM chemistry is critically important to pushing the boundaries of environmental science forward. For example, a present paradigm in environmental science views aerobic respiration rates as being primarily determined by kinetics [i.e., organic carbon (OC) and oxygen concentrations]. However, recent field and laboratory studies suggest that OM thermodynamics can be a main driver of aerobic respiration (Graham et al., 2017, 2018; Stegen et al., 2018b; Garayburu-Caruso et al., 2020). Conventional lumped biogeochemical models are not completely effective for addressing this issue because the thermodynamic properties of substrates are a function of chemical composition of compounds constituting OM pools. This implies that identification of underlying key processes that drive aerobic respiration requires advanced biogeochemical models that can properly reflect all relevant kinetics and thermodynamics in representing actual oxidation rates.

Toward filling this gap, we propose a new modeling concept drawn from thermodynamic theory that can explicitly account for the chemical composition of individual molecules in OM pools when generating biogeochemical rate estimates, therefore having the potential to significantly advance predictive capabilities of current-generation ecosystem models. Due to the ability of our approach to directly incorporate OM chemistry for every compound, we termed it *substrate-explicit modeling* (SXM), as opposed to MXM and EXM. SXM features flexibility in input data types, enabling incorporation of an unlimited number of compounds detected from various state-of-the-art instrumentation including GC-MS, LC-MS/MS, HPLC-MS, NMR, Orbitrap MS, and Fourier transform ion cyclotron resonance (FTICR-MS). Our modeling framework therefore overcomes a central challenging in using such information—how to condense the massive amount of data produced by these technologies into variables that are both useful and computationally feasible in predicting biogeochemical dynamics and function.

By combining a suite of previously developed thermodynamic theories (McCarty, 2007; Kleerebezem and Van Loosdrecht, 2010; LaRowe and Van Cappellen, 2011; Desmond-Le Quemener and Bouchez, 2014), we developed a systematic procedure to convert chemical formulae of all organic compounds detected in an environment, regardless of the number of compounds or measurement technique, into two rate parameters that integrate seamlessly into a variety of modeling constructs. We evaluated the effectiveness of our approach through the analysis of OM characterized via FTICR-MS data obtained from two biogeochemically distinct sites. Model outputs were compared to experimental work on the same samples by Graham et al. (2017, 2018), who showed that aerobic respiration varied between across sites by several-fold range. We chose to model two representative OM profiles from the samples with high (high activity, HA) and low (low activity, LA) rates of aerobic respiration to test model predictions against the broadest range of biogeochemical activity in the dataset.

Consistent with the findings from Graham et al. (2017, 2018), comparative analyses of the two SXMs constructed for HA and LA zones showed that respiration reactions of OM pools in HA zones were thermodynamically more favorable than those in LA zones and that this difference of OM thermodynamic properties was associated with elevated respiration rates in the HA zone. Further comparison of predicted reaction rates and experimentally determined aerobic respiration predicted field conditions as being limited by carbon. Lastly, we showed that SXMs have a flexible structure to be synergistically integrated with other existing frameworks (such as EXMs and/or MXMs) toward more comprehensive predictive biogeochemical modeling. Through coupling with reactive transport models, this hybridized or integrative biogeochemical modeling (IBM) is expected to significantly expand our ability to predict complex ecosystem functions.

## Materials and Methods

**FIGURE 1:**
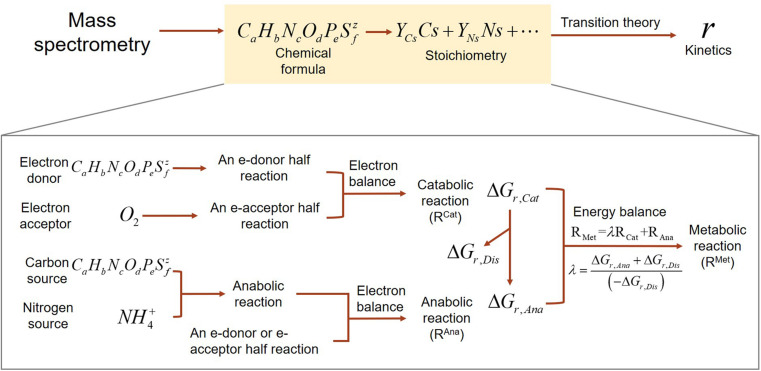
A schematic illustrating the flows of building substrate-explicit models from chemical formulae of OC to stoichiometry and kinetics of oxidative respiration. The zoomed-in box below shows the bottom–up derivation of biogeochemical reactions for each OC.

Our development of SXMs from chemical formula of OC go through multiple-step procedures as illustrated in Figure 1, which are classified into two major parts: (1) derivation of stoichiometric equations for catabolic, anabolic, and metabolic reactions by combining a set of standard thermodynamic analyses (McCarty, 2007; Kleerebezem and Van Loosdrecht, 2010; LaRowe and Van Cappellen, 2011); and (2) formulation of kinetic equations for the final oxidative degradation reaction of OC using a relatively recent thermodynamic theory for microbial growth (Desmond-Le Quemener and Bouchez, 2014).

### Stoichiometric Representation of Oxidative Respiration Reactions

Stoichiometric equations provide quantitative relationships among the reactants and products involved in given reactions. We derived a stoichiometric equation for oxidative degradation of OC by accounting for catabolism (i.e., all processes for obtaining energy through substrate oxidation or other means) and anabolism (i.e., synthesis of biomass using the energy provided from catabolism) (Figure 1). Combination of catabolic and anabolic reactions through energy coupling leads to metabolic reactions. We formulate catabolic reactions (and anabolic reactions as well in many cases) as combinations of a pair of redox half reactions, i.e., for an electron donor (Ed) and an electron acceptor (Ea), while cases exist where catabolism can occur without involving electron donor/acceptor coupling, e.g., in disproportionation reactions. Therefore, oxidative degradation of OC described here as a metabolic reaction can be systematically derived in a bottom–up fashion. We provide step-by-step procedures of developing stoichiometric equations following the standard approaches outlined in the literature (Kleerebezem and Van Loosdrecht, 2010; Rittmann and McCarty, 2012). For a general representation of all associated reactions, we use the following convention for stoichiometric representation:

(1) $$\sum_{i=1}^{n}y_{S_{i}}{\text{S}}_{\text{i}}=0$$

where S_i_ denotes chemical species *i*, *y*_*S_i_*_ (≥0) is the stoichiometric coefficient of S_i_, which is set to be negative for reactants and positive for products. For a defined array of S_i_’s, Eq. (1) can be conveniently represented as a column vector of stoichiometric coefficients:

(2) $$y={\left[y_{1},y_{2},\cdots ,y_{n}\right]}^{\text{T}}$$

where the superscript T denotes the vector transpose. We specified 10 chemical species (S_*i*_’s) involved in OC oxidation reactions (i.e., *n* = 10) and provided standard state Gibbs energy of formation of each compound (Table 1). The standard state of a compound in aqueous phase denotes is unit activity in a hypothetical one molal solution referenced to infinite dilution at any temperature and pressure (Amend and LaRowe, 2019).

**TABLE 1:** Key chemical species that are associated with oxidative degradation of OC and their standard Gibbs energy of formation from the elements ($\Delta G_{i}^{0}$).

*i*	S_*i*_	$\Delta G_{i}^{0}$ [kJ/mol]	*i*	S_*i*_	$\Delta G_{i}^{0}$ [kJ/mol]
1	OC	Dependent on chemical formula	6	HS^−^	12.0
2	H_2_O	−237.2	7	H^+^	0*
3	${\text{HCO}}_{3}^{-}$	−586.9	8	e^−^	0*
4	${\text{NH}}_{4}^{+}$	−79.5	9	O_2_	16.5
5	${\text{HPO}}_{4}^{2-}$	−1089.1	10	Biom	−67**

The standard state values of Gibbs energies of all compounds except biomass (Biom) were calculated using the CHNOSZ R package (Dick, 2019) at 298.15K (25^o^C) and 1 bar. i, index of chemical species; OC, organic carbon; Biom, biomass (CH_1.8_N_0.2_O_0.5_) (Stephanopoulos et al., 1998; Kleerebezem and Van Loosdrecht, 2010). *All thermodynamic properties of H^+^ and e^–^ including Gibbs energies are 0 at all pressures and temperatures and all other aqueous species properties are relative to this.**$\Delta G_{i}^{0}$for Biom was taken from Kleerebezem and Van Loosdrecht (2010).

#### Metabolic Reactions

A stoichiometric equation of metabolic reactions can be derived by accounting for the energetic coupling between catabolism and anabolism, i.e., the balance between the energy production through substrate degradation and the energy consumption for cell synthesis (i.e., biomass production) (see Supplementary Text for derivation of stochiometric equations of catabolic and anabolic reactions). Two approaches commonly considered for this purpose include the dissipation method (Heijnen et al., 1992; Heijnen and Vandijken, 1993) and the thermodynamic electron equivalents model (TEEM) (McCarty, 2007). In this work, we combined the dissipation method and TEEM. We used the dissipation method as a basic framework, which determines the stoichiometric coefficient vector for metabolic reaction (*y*) by coupling the catabolic and anabolic reactions based on the parameter λ, i.e.,

(3) $$y=\lambda y^{Cat}+y^{An}$$

where

(4) $$\lambda =\frac{\Delta G_{r,Ana}+\Delta G_{r,Dis}}{\left(-\Delta G_{r,Cat}\right)}$$

Here, *y*^*Cat*^ and *y*^*An*^ are column vectors of stoichiometric coefficients in catabolic and anabolic reactions, and Δ*G*_*r*,*Cat*_ and Δ*G*_*r*,*Ana*_, respectively, denote Gibbs energies of catabolic and anabolic reactions, and Δ*G*_*r*,*Dis*_ is dissipation energy. The parameter λ implies how many times the catabolic reaction needs to run to provide the energy required for the synthesis of a unit C-mole of biomass. Lower values of λ therefore represent higher thermodynamic favorability of metabolic reactions involving given OM. The value of λ is expected to increase under stress conditions where energy efficiency for growth can become low, e.g., due to increased energy dissipation.

We used TEEM to calculate the value of λ and subsequently dissipation energy (Δ*G*_*r*,*Dis*_) because the formula in the dissipation method (used to calculate Δ*G*_*r*,*Dis*_) is not extendable to complex OCs with more than six-carbon OCs, while most of the compounds profiled in our samples are more complex than those. The TEEM considers the energy provision of catabolic reaction to meet the energy spent in the following two steps of energy conversion in anabolism: (1) the carbon source to biomass building blocks (Δ*G*_*r*,*Block*_), and (2) the conversion of biomass building blocks to biomass (Δ*G*_*r*,*Syn*_). The original formulation includes the energy conversion from the nitrogen source to ammonium, which is neglected in our case where ammonium is taken as the nitrogen source. The energy balance is then represented using the following equation:

(5) $$\lambda \left(\eta \Delta G_{r,Cat}\right)+\eta^{m}\Delta G_{r,Block}+\Delta G_{r,Syn}=0$$

where Δ*G*_*r*,*Cat*_ is the Gibbs free energy change in catabolism, λ is a parameter that couples catabolism and anabolism as defined earlier, and η is an energy transfer efficiency parameter. The exponent *m* accounts for energy transfer efficiency depending on whether energy is generated or consumed in the process of converting the carbon source to biomass building blocks by setting it to +1 (if Δ*G*_*r*,*Block*_ < 0) or −1 (if Δ*G*_*r*,*Block*_ > 0). Following Kleerebezem and Van Loosdrecht (2010), we chose Δ*G*_*r*,*Syn*_ = 200kJ/(molBiom), and η = 0.43; assumed the composition of biomass building block to be the same as Biom (i.e., CH_1.8_N_0.2_O_0.5_). Constant composition of biomass and building block means thatΔ*G*_*r*,*Block*_ is the same across samples. Specific biomass composition used in this work (CH_1.8_N_0.2_O_0.5_) has been widely used in the literature (Stephanopoulos et al., 1998; Kleerebezem and Van Loosdrecht, 2010), while experimental determination would enhance the calculation of Gibbs energies (LaRowe and Amend, 2016).

Determination of λ from Eq. (5) then requires the calculation of the Gibbs free energy Δ*G*_*r*,*Block*_, which is assumed to be the same as Δ*G*_*r*,*Ana*_ in our formulation (where the elemental composition of biomass building block was treated as the same as Biom). With the value of λ calculated as such, one can now determine the value of Δ*G*_*r*,*Dis*_ from Eq. (4) and the full stoichiometric equation of metabolic reaction from Eq. (3). In the following section, we describe how to calculate the Gibbs free energy changes in a general context.

### Gibbs Free Energy Change

The Gibbs free energy change of a reaction (Δ*G*_*r*_) can be calculated from

(6) $$\Delta G_{r}=\Delta G_{r}^{0}+RT\ln Q_{r}$$

where $\Delta G_{r}^{0}$ denotes the standard state Gibbs energy of a reaction, *R* is the gas constant [= 0.008314 kJ/(K ⋅ mol)], *T* is temperature in Kelvin, and *Q*_*r*_ is the reaction quotient defined by

(7) $$Q=\underset{i}{\Pi }{\left(a_{i}\right)}^{y_{i}}$$

where *a*_*i*_ and *y*_*i*_ denote the activity and stochiometric coefficient of the *i*^*th*^ chemical species.

For aqueous systems, Gibbs free energy of a reaction is often calculated for the standard state where substances take unit activities (i.e., ln *Q*_*r*_ = 0 and therefore Δ*G*_*r*_ reduces to $\Delta G_{r}^{0}$) in a hypothetical one molal solution referenced to infinite dilution. Standard state Gibbs energies of a reaction ($\Delta G_{r}^{0}$) can be calculated based on standard state Gibbs energy of formation of all associated compounds ($\Delta G_{i}^{0}$) and their stoichiometry (*y*_*S_i_*_), i.e.,

(8) $$\Delta G_{r}^{0}=\sum_{i=1}^{n}y_{S_{i}}\Delta G_{i}^{0}$$

As the standard state assuming one molal (i.e., unity activities) of aqueous species (i.e., thus pH = 0) does not properly represent the state of biological cells, one can also define the biological standard state where pH is set to 7 (i.e., $a_{H^{+}}=10^{-7}$). The biological standard state (denoted as by $\Delta G_{r}^{0^{\prime }}$) is then calculated by

(9) $$\Delta G_{r}^{0^{\prime }}=\Delta G_{r}^{0}+RTy_{H^{+}}\ln\left(10^{-7}\right)$$

The above equation defines the biological standard state Gibbs energies for most cells (neutrophils), while acidophiles and alkaliphiles optimally grow at lower and higher pH than 7 (Amend and LaRowe, 2019; Keenleyside, 2019). The use of $\Delta G_{r}^{0^{\prime }}$is generally regarded acceptable for thermodynamic analysis of redox reactions in biochemical systems involving oxidative respiration (Kleerebezem and Van Loosdrecht, 2010).

For all compounds considered in this work except OC, the values of $\Delta G_{i}^{0}$ in Eq. (8) are readily obtainable from public databases or the literature (see Table 1) (Kleerebezem and Van Loosdrecht, 2010; Dick, 2019). Theoretical estimation of the standard formation energy of OC ($\Delta G_{OC}^{0}$) using the group contribution theory is infeasible because the structural information of compounds is not available in FTICR-MS. Therefore, we estimated $\Delta G_{OC}^{0}$ in the following way.

First, we estimated the standard Gibbs free energy change for an electron donor half reaction ($\Delta G_{r,D}^{0}$) using the formula from LaRowe and Van Cappellen (2011). They provided a linear relationship between the nominal oxidation state of carbon (NOSC) and the Gibbs energies for the oxidation half reactions of OC represented on a C-mole basis ($\Delta G_{Cox}^{0}$), i.e.,

(10) $$\Delta G_{Cox}^{0}\left[\mathrm{kJ}/C-\mathrm{mol}\right]=60.3-28.5\text{NOSC}$$

where NOSC is obtained from the exchanged electron moles in the half reaction, and the number of carbon in OC:

(11) $$\text{NOSC}=-\left(n_{e^{-}}/a\right)+4$$

where *n*_*e*^–^_ is the number of electrons transferred in the half reaction ($=y_{e^{-}}$) and a denotes the number of C elements in OC. As $\Delta G_{Cox}^{0}$[kJ/C-mol] represents the Gibbs free energy per one C-mole, $\Delta G_{r,D}^{0}$ is obtained simply by multiplying the number of carbon in OC, i.e.,

(12) $$\Delta G_{r,D}^{0}\left[\mathrm{kJ}/\mathrm{mol}\right]=a\Delta G_{Cox}^{0}$$

Then, the substation of $\Delta G_{r,D}^{0}$ in the above into Eq. (8) leads to

(13) $$\Delta G_{OC}^{0}=\left(\Delta G_{r,D}^{0}-\sum_{i=2}^{10}y_{i}^{D}\Delta G_{i}^{0}\right)/y_{OC}^{D}$$

where $\Delta G_{r,D}^{0}$ is standard Gibbs energy of an electron-donor half reaction, $y_{i}^{D}$ denotes the stoichiometric coefficient of the *i*^*th*^ compound in an electron-donor half reaction, and $y_{OC}^{D}=-1$ in our formulation (see Supplementary Text for the formulation of an electron donor half reaction). Once $\Delta G_{OC}^{0}$ is known from Eq. (13), it is straightforward to calculate the standard (at pH = 0) and biochemical standard (at pH = 7) Gibbs free energy changes (i.e., $\Delta G_{r}^{0}$ and $\Delta G_{r}^{0^{\prime }}$) for any given reactions. Hereafter, we drop the superscript 0′ to denote thermodynamic functions at pH = 7, while we still keep the superscript 0 to denote their values at pH = 0.

### Reaction Kinetics

Thermodynamic theory by Desmond-Le Quemener and Bouchez (2014) enables formulating microbial growth kinetics from stoichiometric equations derived in the previous section (see also Supplementary Text for derivation). In the case of oxidative degradation of OC, the microbial growth on the *i*^*th*^ OC (OC_*i*_) can be represented by

(14) $$\mu_{i}=\mu^{\max}\exp\left(-\frac{\left|y_{OC,i}\right|}{V_{h}\left[{\text{OC}}_{i}\right]}\right)\exp\left(-\frac{\left|y_{O_{2},i}\right|}{V_{h}\left[{\text{O}}_{2}\right]}\right)$$

where μ^max^ is the maximal specific growth rate, *V*_*h*_ is the volume that a microbe can access for harvesting energy from the environment (thus termed harvest volume), *y*_*OC,i*_ and *y*_*O*_2_,*i*_ are the stoichiometric coefficients of OC and O_2_ in the metabolic reaction associated with oxidative degradation of OC_i_, and |*y*_*OC*,*i*_| and |*y*_*O*_2_,*i*_| denote their absolution values. Through comparison with Michaelis–Menten (M–M) kinetics, Desmond-Le Quemener and Bouchez (2014) showed that this non-conventional functional form in Eq. (14) (as well as M–M kinetics) can provide a quantitative fit to experimental data.

The above equation shows the case when both C and O_2_ are limited. If only C or O_2_ is limited, Eq. (14) is reduced to

(15) $$\mu_{i}=\left\{ \begin{array}{c} \mu^{\max}\exp\left(-\frac{\left|y_{OC,i}\right|}{V_{h}\left[{\text{OC}}_{i}\right]}\right),\mathrm{OC}\text{-}\mathrm{limited}\mathrm{in}\mathrm{excessive}\mathrm{supply}\mathrm{of}{\text{O}}_{2}\\ \mu^{\max}\exp\left(-\frac{\left|y_{O_{2},i}\right|}{V_{h}\left[{\text{O}}_{2}\right]}\right),{\text{O}}_{2}\text{-}\mathrm{limited}\mathrm{in}\mathrm{excessive}\mathrm{supply}\mathrm{of}\text{OC} \end{array}\right.$$

Consumption and production rates of other chemicals (such as OC, C, O_2_, and ${\text{HCO}}_{3}^{-}$) can be obtained simply by multiplying stoichiometric coefficients and μ_*i*_in Eq. (14) or (15), i.e.,

(16a) $$r_{OC,i}=y_{OC,i}\mu_{i}$$

(16b) $$r_{O_{2},i}=y_{O_{2},i}\mu_{i}$$

(16c) $$r_{HCO_{3}^{-},i}=y_{HCO_{3}^{-},i}\mu_{i}$$

Finally, it is straightforward to set up dynamic mass balances. For example, in a homogeneous batch configuration, dynamic SXMs for key metabolites that are associated with oxidative respiration of OC_i_ can be written as follows:

(17a) $$\frac{d\left[{\text{OC}}_{i}\right]}{dt}=r_{OC,i}\left[\text{B}\right]$$

(17b) $$\frac{d\left[{\text{O}}_{2}\right]}{dt}=r_{O_{2},i}\left[\text{B}\right]$$

(17c) $$\frac{d\left[{\text{HCO}}_{3}^{-}\right]}{dt}=r_{HCO_{3}^{-},i}\left[\text{B}\right]$$

(17d) $$\frac{d\left[\text{B}\right]}{dt}=\mu_{i}\left[\text{B}\right]$$

### Integration With MXM and EXM

The SXM derived in Eq. (17) can be combined with other complementary approaches. Integration of SXM with MXM requires explicit consideration of distinct microbial groups, which leads to

(18a) $$\frac{d\left[{\text{OC}}_{i}\right]}{dt}=\sum_{j}r_{OC,i,j}\left[{\text{B}}_{j}\right]$$

(18b) $$\frac{d\left[{\text{O}}_{2}\right]}{dt}=\sum_{j}r_{O_{2},i,j}\left[{\text{B}}_{j}\right]$$

(18c) $$\frac{d\left[{\text{HCO}}_{3}^{-}\right]}{dt}=\sum_{j}r_{HCO_{3}^{-},i,j}\left[{\text{B}}_{j}\right]$$

(18d) $$\frac{d\left[\text{B}\right]}{dt}=\sum_{j}\mu_{i,j}\left[{\text{B}}_{j}\right]$$

where the subscript *j* denotes the contribution of the *j*^*th*^ microbial group to the production and consumption of metabolites, and μ_*i,j*_ is the growth rate of the *j*^*th*^ microbial group on *OC*_*i*_, i.e.,

(16) $$\mu_{i,j}=\mu_{j}^{\max}\exp\left(-\frac{\left|y_{OC,i}\right|}{V_{h}\left[{\text{OC}}_{i}\right]}\right)\exp\left(-\frac{\left|y_{O_{2},i}\right|}{V_{h}\left[{\text{O}}_{2}\right]}\right)$$

Note that this formulation accounts for the difference in growth rate among microbial groups. Production and consumption rates of metabolites are accordingly dependent on microbial groups, i.e.,

(20a) $$r_{OC,i,j}=y_{OC,i}\mu_{i,j}$$

(20b) $$r_{O_{2},i,j}=y_{O_{2},i}\mu_{i,j}$$

(20c) $$r_{HCO_{3}^{-},i,j}=y_{HCO_{3}^{-},i}\mu_{i,j}$$

For the integration of SXM with EXM, we introduce enzyme concentrations as additional variables. Dynamic mass balances in Eq. (17) remain the same, but we consider individual reactions to be catalyzed by distinct enzymes, i.e.,

(17) $$\mu_{i}=e_{i}^{rel}\mu_{i}^{kin}$$

where the reaction rate (μ_*i*_) is composed of two parts: enzymatic regulation ($e_{i}^{rel}$) and kinetic conversion ($\mu_{i}^{kin}$), and $e_{i}^{rel}$denotes relative level of enzyme, i.e.,

(18) $$e_{i}^{rel}=\frac{e_{i}}{e_{i}^{\max}}$$

Enzyme concentrations and their maximal values can be determined from the following equation:

(19) $$\frac{de_{i}}{dt}=\alpha_{i}+u_{i}r_{E,i}^{kin}-\beta_{i}$$

where three terms on the right hand side of the above equation respectively denote constitutive enzyme synthesis rate, inductive enzyme synthesis rate, and enzyme degradation rate, *u*_*i*_ is the variable that controls inductive enzyme synthesis, and $r_{E,i}^{kin}$ is the kinetic rate of enzyme synthesis. We formulated the control variable *u*_*i*_ based on the cybernetic modeling approach (Ramkrishna and Song, 2012, 2018), which views microorganisms as a dynamic control system that maximizes a given metabolic objective (such as biomass production rate) through optimal synthesis of enzymes. The cybernetic model has been successfully applied to simulate complex microbial growth patterns on multiple nutrients and electron acceptors (Song and Liu, 2015; Song et al., 2017). Instead of solving separate enzyme equations, we considered a simplified form of cybernetic model (Song et al., 2018) where $e_{i}^{rel}$ in Eq. (17) is approximated by *u*_*i*_ (Song and Ramkrishna, 2010, 2011), which is in turn determined based on $\mu_{i}^{kin}$. Then, Eq. (17) becomes

(20) $$\mu_{i}=u_{i}\mu_{i}^{kin}$$

where

(21) $$u_{i}=\frac{\mu_{i}^{kin}}{\sum_{j}\mu_{j}^{kin}}$$

### Field Samples

To demonstrate our modeling concept described above, we used the datasets generated in a previous work, described in detail in Graham and colleagues (Graham et al., 2017, 2018). Briefly, sediment profiles (0–60 cm) were collected along two shoreline transects perpendicular to the Columbia River within the Hanford Site 300 Area in eastern Washington State (Graham et al., 2016, 2017; Slater et al., 2010; Zachara et al., 2013): one with riparian vegetation (HA zone) and the other without (LA zone). In each transect, profiles were collected from three locations: upper, mid, and lower banks, and sectioned into 10 cm vertical intervals. FTICR-MS was used to characterize C chemistry in each sample, and Raz reduction assay was performed as a proxy of the rate of aerobic respiration per sample. For each peak detected in FTICR-MS spectra, we assigned a chemical formula using the following steps: (1) transformation of raw spectra to m/z (i.e., mass divided by charge number) values using BrukerDaltonik software; (2) chemical formula assignment using in house software following the Compound Identification Algorithm (Kujawinski and Behn, 2006; Minor et al., 2012; Tfaily et al., 2017). In the case that one m/z value can be matching with multiple chemical formulae, consistent assignment was made based on a set of prescribed rules, including the preference of the formula with the lowest error and with the lowest number of heteroatoms and the requiring the presence of at least four oxygen atoms for the assignment of one phosphorus atom. Peaks not satisfying these criteria were not assigned chemical formulae.

For detailed analysis and comparison of two sites, we chose one sectioned sample from the LA and HA profiles: (1) Upper bank at unvegetated site (N1), 40–50 cm depth interval (sample N1-40-50) and (2) Upper bank at vegetated site (S1), 0–10 cm depth interval (sample S1-00-10). These two samples respectively represent the lowest and highest activity in aerobic respiration except an outlier (see section “Results: Comparison of Low-Activity and High-Activity Samples”).

## Results

### Evaluation of Thermodynamic Parameters as an Indicator of Oxidative Respiration

**FIGURE 2:**
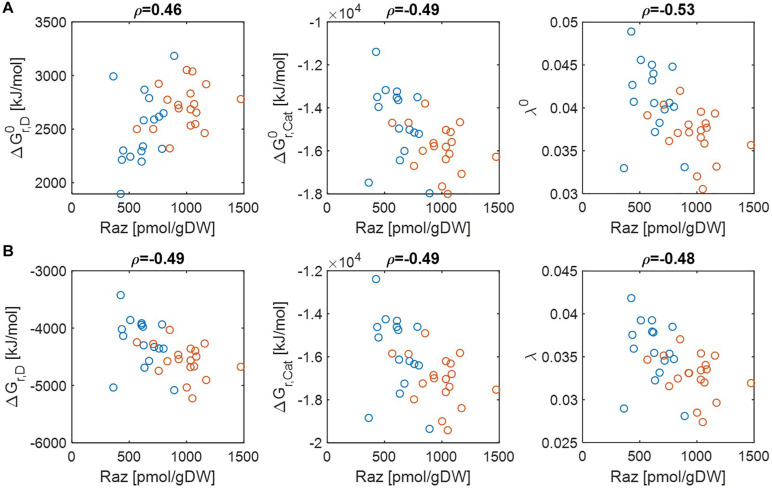
Pearson correlations (*ρ*) of aerobic respiration with three key thermodynamic functions: standard Gibbs free energy changes for an electron donor half reaction (Δ*G*_*r*,*D*_) and catalytic reaction (Δ*G*_*r*,*Cat*_), and the energy coupling parameter (λ): **(A)** pH = 0 (i.e., activity of hydrogen ion (*a*_*H*^+^_) = 1 (denoted by the superscript 0) and **(B)** pH = 7 (i.e., $a_{H^{+}}=10^{-7}$). The values of standard state of Gibbs energies were calculated at 25°C and 1 bar. Raz denotes the amount of resazurin reduced to resorufin over the 48 h incubation period. Blue and orange open circles denote the samples from low and high activity zones.

Previous studies have shown that aerobic respiration rates from HA samples were higher than LA samples and provided the hypothesis that thermodynamic properties of OM might be a key factor in regulating respiration rate (Graham et al., 2017, 2018). While the original papers provided datasets that convincingly support this hypothesis, the question of what thermodynamic parameters (among many introduced in section “Materials and Methods”) can serve as reliable indicators of aerobic respiration remains unknown. We therefore chose multiple thermodynamic parameters and examined to what extent individual parameters are correlated with aerobic respiration. Three key parameters include Gibbs free energy changes for electron donor half reactions (Δ*G*_*r*,*D*_), Gibbs free energy changes for catabolic reactions (Δ*G*_*r*,*Cat*_), and the parameter quantifying the energy coupling between catabolic and anabolic reactions (λ). The first two parameters (Δ*G*_*r*,*D*_ and Δ*G*_*r*,*Cat*_) evaluate the thermodynamic character of OM with a focus on the energy *generation* (through a half or complete catabolic reaction, respectively). By contrast, the parameter λ quantifies the same based on the energy *balance* (i.e., the total energy generation to meet the demand for the synthesis of a unit C-mole of biomass). Therefore, all of these three parameters measure thermodynamic *inefficiency* of OM (in the sense that lower values of parameters imply higher thermodynamic efficiencies of OM), but at different levels, i.e., half/complete catabolic reactions for Δ*G*_*r*,*D*_ and Δ*G*_*r*,*Cat*_, and entire metabolic reaction for λ. We examined their correlations with experimentally determined aerobic respiration at pH = 0 and pH = 7 (Figure 2). We also examined whether the thermodynamic favorability of compound can be better characterized on a C-mole or OM-mole basis (Supplementary Figure S1).

Regardless of pH values, Δ*G*_*r*,*Cat*_ and λ showed negative correlations with aerobic respiration, which implies that thermodynamic properties of OM lead to the difference in respiration rates (the middle and right panels in Figures 2A,B). By contrast, the results for Δ*G*_*r*,*D*_ were pH-dependent, i.e., Δ*G*_*r*,*D*_ showed a positive correlation with aerobic respiration at pH = 0 (the left panel of Figure 2A) (indicating that Δ*G*_*r*,*D*_ is not a good estimator of aerobic respiration under this condition), while it showed a negative correlation at pH = 7 (the left panel of Figure 2B).

As C-mole, the parameter λ still showed negative correlations with aerobic respiration at both pH values (Supplementary Figure S1). Δ*G*_*r*,*D*_ and Δ*G*_*r*,*Cat*_ showed weak relationships with aerobic respiration with correlation coefficients between −0.1 and 0.1, except for Δ*G*_*r*,*D*_ at pH = 7 where the correlation coefficient was positive.

Together, these results identify the parameter λ as the most robust indicator of aerobic respiration, which shows consistent correlations across all conditions considered above. The results also indicate that the usefulness of the other thermodynamic parameters (Δ*G*_*r*,*D*_ and Δ*G*_*r*,*Cat*_) as a respiration indicator is relatively limited.

### Model Validation by Comparing Predicted Reaction Rates With Aerobic Respiration

Comparison of predicted reaction rates with experimentally determined aerobic respiration provides a means to validate the model. The experiment of Raz reduction assay in Graham and colleagues (Graham et al., 2017, 2018) was designed as a proxy measurement for the rate of oxygen consumption. For validation, we checked to what degree our model can predict oxygen consumption rates that are positively correlated with experimental estimation. As reaction rates are functions of substrate concentrations, we performed this comparison under three different limiting conditions: (1) C, (2) O_2_, and (3) both C&O_2_ limitation. For each, we differentiated the level of limitation to be severe vs. moderate.

**FIGURE 3:**
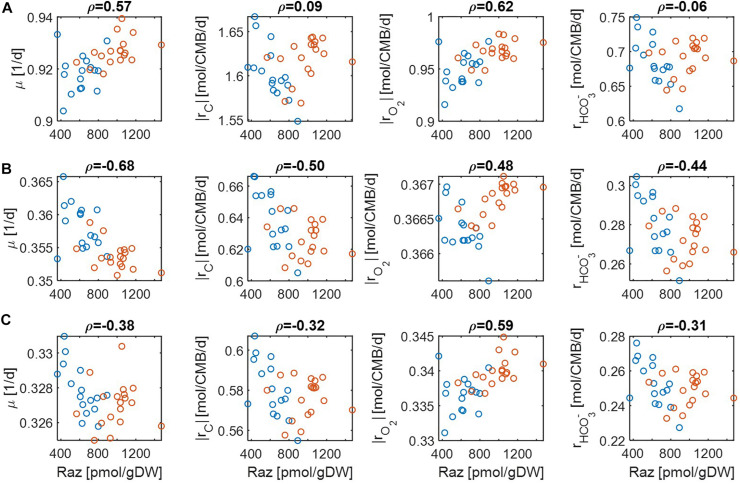
Pearson correlations (*ρ*) of aerobic respiration with predicted reaction rates under moderate nutrient limitations (i.e., *V*_*h*_[*OC*] = 1 and/or *V*_*h*_[*O*_2_] = 1): growth rate (μ), carbon consumption rate (|*r*_*C*_|), O_2_ consumption rate (|*r*_*O*_2__|), and bicarbonate production rate ($r_{HCO_{3}^{-}}$): **(A)** C-limited condition, **(B)** O_2_-limited condition, and **(C)** both C&O_2_-limited condition. Raz denotes the amount of resazurin reduced to resorufin over the 48hr incubation period. Blue and orange open circles denote the samples from low and high activity zones.

**FIGURE 4:**
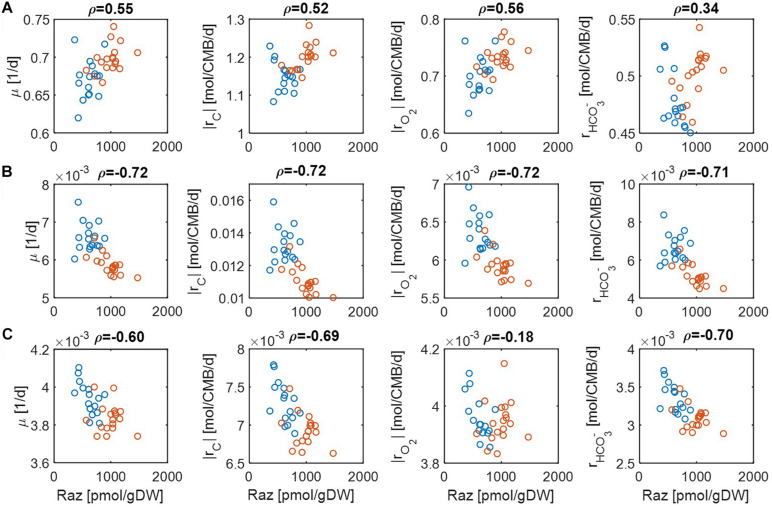
Pearson correlations (*ρ*) of aerobic respiration with predicted reaction rates under severe nutrient limitations (i.e., *V*_*h*_[*OC*] = 0.2 and/or *V*_*h*_[*O*_2_] = 0.2): growth rate (μ), carbon consumption rate (|*r*_*C*_|), O_2_ consumption rate (|*r*_*O*_2__|), and bicarbonate production rate ($r_{HCO_{3}^{-}}$): **(A)** C-limited condition, **(B)** O_2_-limited condition, and **(C)** both C&O_2_-limited condition. Raz denotes the amount of resazurin reduced to resorufin over the 48 h incubation period. Blue and orange open circles denote the samples from low and high activity zones.

For simplicity, we set μ^max^ = 1 in Eqs. (14) and (15) because this value does not affect the correlation with aerobic respiration, while other parameters and variables such as *V*_*h*_, [OC], and [O_2_] are unknown. To implement different levels of substrate limitation, we set *V*_*h*_[OC] and/or *V*_*h*_[O_2_] to be 1 (moderate limitation) (Figure 3) and 0.2 (severe limitation), respectively (Figure 4) in the following four reaction rates: biomass production (i.e., growth) rate (μ), C consumption rate [*r*_*C*_ ≡ (# of C) × *r*_*OC*_], O_2_ consumption rate (*r*_*O*_2__), and inorganic carbon production rate ($r_{HCO_{3}^{-}}$).

For the case of moderate substrate limitation (Figure 3), for example, predicted μ showed a positive correlation under the C-limited condition (meaning that microbial growth rate is increasing with respiration rate), which was however turned into negative correlations under O_2_− or both C&O_2_-limited conditions (meaning that microbial growth rate decreases as respiration rate increases). Similar patterns were observed for |*r*_*C*_| (i.e., the absolute value of *r*_*C*_) and $r_{HCO_{3}^{-}}$, while their correlations with aerobic respiration were weak under the C-limited condition. As an exception, the correlation of |*r*_*O*_2__| with aerobic respiration was consistently positive across all three different limiting conditions. As mentioned above, this result partially validates our model because Raz reduction to resorufin represents an estimate of the consumption rate of O_2_, rather than other chemicals (Gonzalez-Pinzon et al., 2012).

The results above were significantly changed when substrate limitation was severe (Figure 4). In this case, correlation patterns among four different reaction rates were shown to be similar. That is, they all showed positive correlations with aerobic respiration under C-limited conditions and negative correlations under O_2_− and both C&O_2_-limited conditions. Interestingly, the positive correlation of aerobic respiration under C-limited conditions was the highest for microbial growth and O_2_ consumption rates. Together with the previous result, this shows that experimental data in Graham et al. (2017) may be consistently interpreted as being C-limited.

### Comparison of Low-Activity and High-Activity Samples

To understand how biogeochemistry in low- and high-activity zones is differentiated, we performed detailed analyses of two selected samples: one from the low-activity zone (sample N1-40-50) and the other from the high-activity zone (sample S1-00-10) (see section “Materials and Methods”). With outliers removed, these two samples represent the highest and lowest activity as shown in the correlations of aerobic respiration with λ and |*r*_*O*_2__| (Supplementary Figure S2).

**FIGURE 5:**
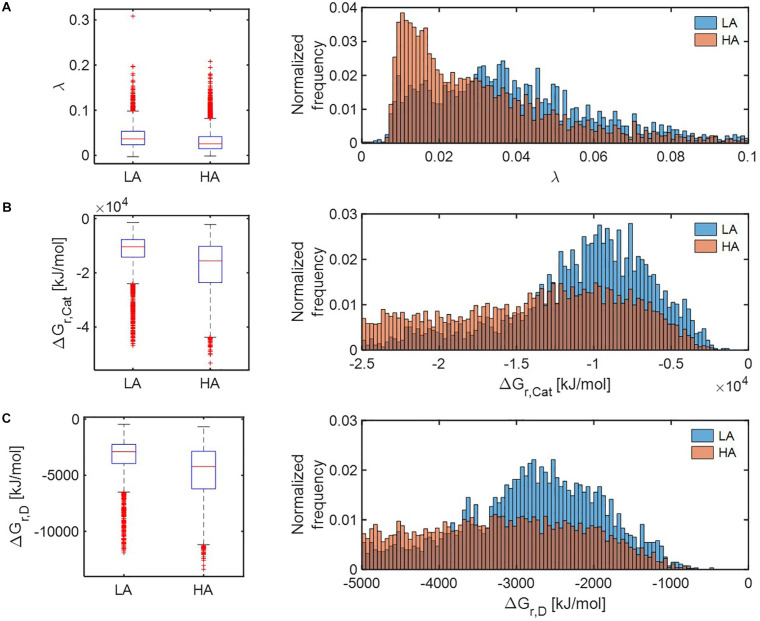
Box plots (left) and histograms (right) for distributions of three key thermodynamic functions: **(A)** the energy coupling parameter (λ), **(B)** Gibbs free energy change for catabolic reaction (Δ*G*_*r*,*Cat*_), and **(C)** Gibbs free energy change for an electron donor half reaction (Δ*G*_*r*,*D*_). LA, low activity zone; and HA, high activity zone.

Distributions of all three thermodynamic parameters (λ, Δ*G*_*r*,*Cat*_, and Δ*G*_*r*,*D*_) indicated that respiration reactions of the OM pools at HA are thermodynamically more favorable than those at LA (Figure 5). The distributions of parameter λ in the HA and LA samples, respectively, showed an exponential decay and bell-shaped patterns, indicating that the HA sample contained a predominantly large portion of OM, metabolic reactions of which are thermodynamically more favorable in comparison to the LA sample (Figure 5A). By contrast, the distributions of Δ*G*_*r*,*Cat*_ (Figure 5B) and Δ*G*_*r*,*D*_ (Figure 5C) suggested that the HA sample contained a lower portion of OM, catabolic and half reactions of which are less favorable compared to the LA sample.

**FIGURE 6:**
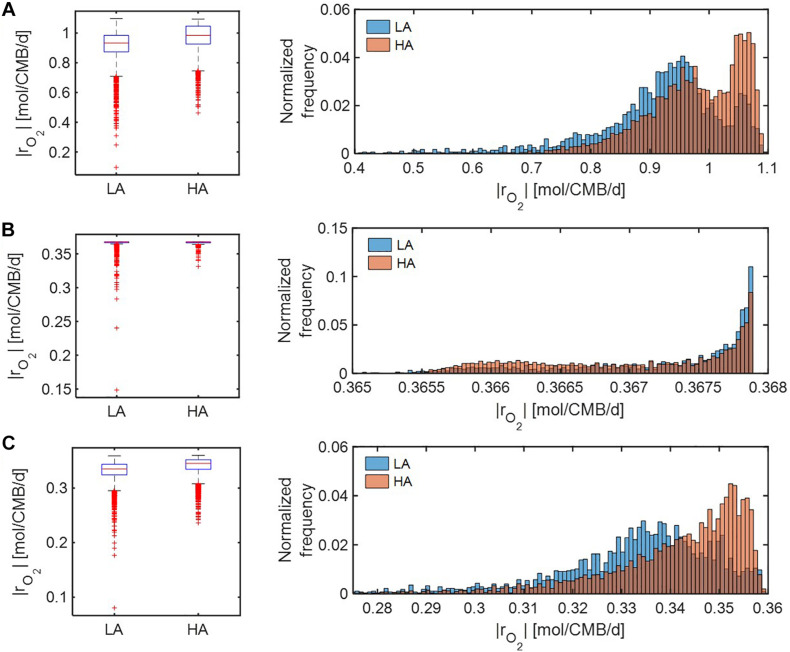
Box plots (left) and histograms (right) for distributions of oxidative respiration rate (|*r*_*O*_2__|) under moderate nutrient limitations (i.e., *V*_*h*_[*OC*] = 1 and/or *V*_*h*_[*O*_2_] = 1): **(A)** C-limited condition, **(B)** O_2_-limited condition, and **(C)** both C&O_2_-limited condition. LA, low activity zone; and HA, high activity zone.

We further compared distributions of model-predicted oxidative respiration rates (|*r*_*O*_2__|) at LA vs. HA under C-, O_2_-, and both C&O_2_-limited conditions. In the case that substrates were *moderately* limited (i.e., *V*_*h*_[*OC*] = 1 and/or *V*_*h*_[*O*_2_] = 1) (Figure 6), predicted oxidative respiration at HA was higher than at LA, when C or both C&O_2_ were limited (as indicated by higher portions of faster |*r*_*O*_2__| in the HA distribution) (Figures 6A,C); however, when O_2_ was limited, there was no significant difference between the two samples (Figure 6B).

**FIGURE 7:**
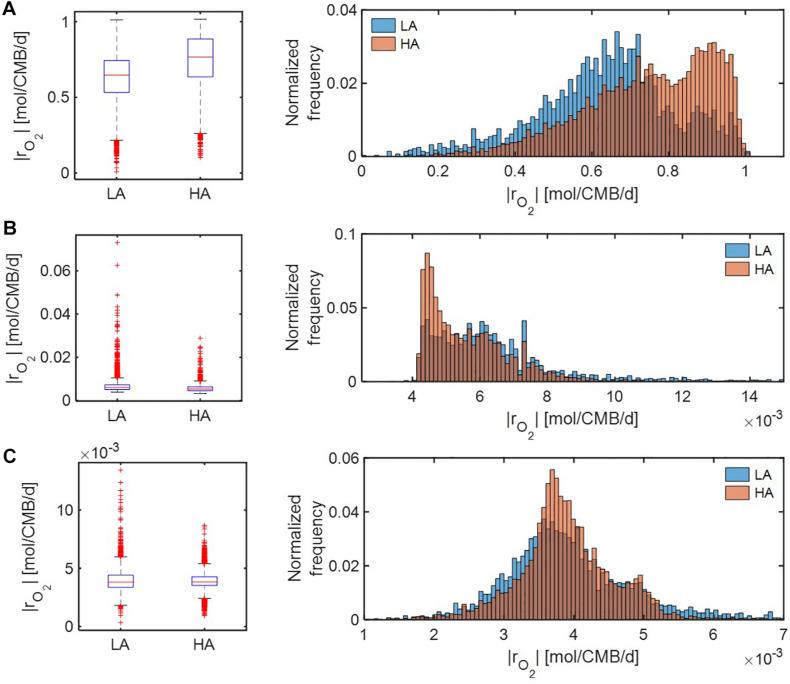
Box plots (left) and histograms (right) for distributions of oxidative respiration rate (|*r*_*O*_2__|) under severe nutrient limitations (i.e., *V*_*h*_[*OC*] = 0.2 and/or *V*_*h*_[*O*_2_] = 0.2): **(A)** C-limited condition, **(B)** O_2_-limited condition, and **(C)** both C&O_2_-limited condition. LA, low activity zone; and HA, high activity zone.

Under *severe* substrate limitation (i.e., *V*_*h*_[OC] = 0.2 and/or *V*_*h*_[O_2_] = 0.2) (Figure 7), predicted oxidative respiration was higher at HA and at LA when C was limited (Figure 7A), while the increase in oxidative respiration at HA was moderate when O_2_ or C&O_2_ were limited (Figures 7A,B). Interestingly, when C was limited, |*r*_*O*_2__| showed bimodal distributions in both HA and LA samples, indicating the non-linear relationship between thermodynamic parameters to reaction rates.

**FIGURE 8:**
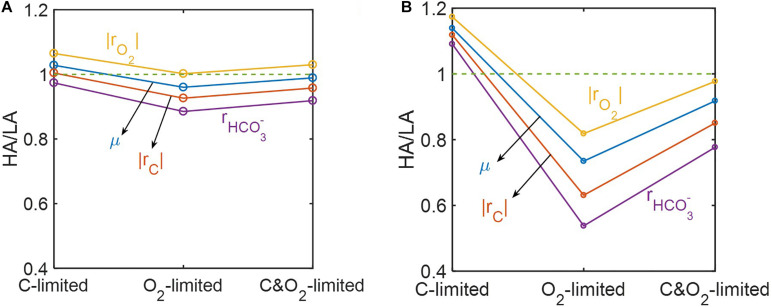
Reaction ratios between the high activity zone (HA) and low activity zone (LA) samples under C-, O_2_-, and C&O_2_-limited conditions: **(A)** moderately limited (i.e., *V*_*h*_[*OC*] = 1 and/or *V*_*h*_[*O*_2_] = 1), and **(B)** severely limited (i.e., *V*_*h*_[*OC*] = 0.2 and/or *V*_*h*_[*O*_2_] = 0.2).

Ratios of predicted reaction rates between the two samples (*HA*/*LA*) showed the level of elevated oxidative respiration in the HA zone (Figure 8). Although these ratios varied among reaction rates and were affected by the level of substrate limitation, their trends across three different substrate-limited conditions were consistent. That is, in cases of moderate (Figure 8A) and severe (Figure 8B) substrate limitation, we consistently found: (1) that the ratios of reaction rates were lowest under O_2_ limitation, intermediate when both C and O_2_ were limited, and highest under C limitation; and (2) that the magnitude of the ratio always maintained the following order: $\left|r_{O_{2}}\right|>\mu >\left|r_{C}\right|>r_{HCO_{3}^{-}}$ (where |*r*_*O*_2__| = respiration rate; μ = microbial growth rate; |*r*_*C*_| = carbon consumption rate; $r_{HCO_{3}^{-}}$ = bicarbonate production rate).

**FIGURE 9:**
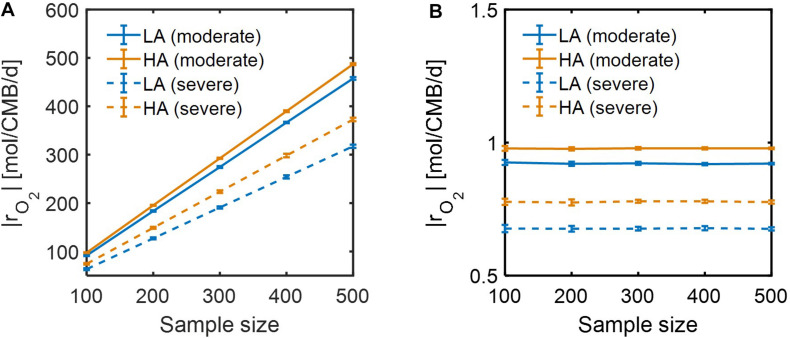
Dependence of simulated respiration rates (|*r*_*O*_2__|) using: **(A)** SXM with no consideration of enzymatic regulation, and **(B)** SXM with enzymatic regulation. The sample size denotes the number of compounds randomly chosen from samples for calculating specific respiration rate per a unit C mole of biomass (CMB).

Lastly, we performed dynamic simulations of OM consumption in LA and HA using two types of models: (1) SXM, and (2) a hybrid model that integrates SXM and EXM. The latter is a special case of a general IBM platform that hybridizes three complementary modeling platforms approaches: SXM-EXM-MXM. In hybridizing SXM with EXM, we used the cybernetic approach (Ramkrishna and Song, 2012, 2018) to account for regulation of enzyme synthesis to simulate selective activation of oxidative reactions among a number of pathways (see section “Materials and Methods”), a key aspect that was missing in the case of using the SXM alone. Although both SXM and hybrid models correctly predicted that specific oxidative reaction rates at HA were higher than at LA (Supplementary Figure S3), the time scale of the SXM was shown to be very small, which led specific reaction rates to be unrealistically high (i.e., more than 300 mol/CMB/d) (Supplementary Figures S3a,b). By contrast, the hybrid model predicted specific reaction rates consistent with the literature values (Supplementary Figures S3c,d). We further compared the two models with respect to how specific OM consumption rates would change with the number of incorporated compounds. As *specific* rates are defined per unit C-mole of biomass, their dependency on the number of compounds is expected to be insignificant, but specific rates calculated by the SXM linearly increased with the number of compounds (Figure 9A), while the hybrid model predicted specific rates to be almost constant regardless of the number of compounds (Figure 9B). These results together indicate that SXM-EXM coupling significantly improved model predictions.

## Discussion

In this work, we proposed a novel and flexible biogeochemical modeling concept, termed SXM. As a key feature, SXM uses thermodynamics to incorporate data from increasingly high-resolution metabolomics technologies into biogeochemical models by formulating OM-specific reaction kinetics for an unlimited number of organic compounds in a sample. The entire set of the resulting reaction kinetics is then represented by only two parameters (i.e., maximal growth rate and harvest volume). Our framework is a unique and scalable tool for modeling complex biogeochemical cycles at the ecosystem-scale, as no other approach can describe dynamic biogeochemical reaction networks composed of thousands of compounds with a small, computationally feasible set of parameters.

The substrate-explicit model is built upon recent experimental studies that reveal a close relationship between OM thermodynamics and aerobic respiration (Graham et al., 2017, 2018; Garayburu-Caruso et al., 2020). In consistent with the conclusions of these studies, our test cases showed that aerobic respiration was driven by thermodynamic favorability of metabolic reactions involving OM pools (as shown by the correlation of measured respiration rates with a thermodynamic parameter λ). These results together challenge classical theories that use the concentrations of bulk substrate pools (such as organic carbon and oxygen) as the sole driving factors of aerobic respiration, notably excluding the influence of OM chemistry on biogeochemistry, highlighting the criticality of incorporation of OM thermodynamics into our understanding and modeling of aerobic respiration.

In this regard, it has been customary to use standard state Gibb energies ($\Delta G_{r}^{0}$) in accounting for thermodynamics in biogeochemical modeling, but improved calculation of thermodynamic functions requires accounting for realistic non-standard conditions (i.e., activities of aqueous species deviating from one molal) (Larowe and Amend, 2015; Amend and LaRowe, 2019). While accurate values of actual Gibbs energies can be obtained by rigorous consideration of activities of all associated chemical species through the reaction quotient term *Q*_*r*_, we used thermodynamic functions corrected to the biological standard state ($\Delta G_{r}^{0^{\prime }}$) (i.e., pH = 7) as a partial remedy in the absence of information on activities of compounds other than H^+^. As a result, $\Delta G_{r}^{0^{\prime }}$ (which is denoted as Δ*G*_*r*_ without the superscript in this article) provided the more interpretable results than $\Delta G_{r}^{0}$. $\Delta G_{Cox}^{0}$[kJ/C-mol] is one of the most frequently used metrics to evaluate thermodynamic favorability of compounds. However, our model suggests λ as a new thermodynamic parameter to evaluate thermodynamic character of OM. In contrast with $\Delta G_{Cox}^{0}$, which considers energy generation only through an electron donor half reaction, λ accounts for the energy balance between catabolic and anabolic reactions, thus evaluating thermodynamic properties of compounds based on the complete chemistry. Indeed, λ consistency showed negative correlations with aerobic respiration, regardless of pH values.

Our model was validated through a consistency check between predicted oxygen consumption rates with experimentally determined aerobic respiration. Across all three conditions (C, O_2_ and, both C&O_2_ limitation), aerobic respiration showed positive correlations with oxygen consumption rate when substrates are moderately limited. By contrast, in the case of severe C and O limitation, a positive correlation between oxygen consumption rate and aerobic respiration was obtained only for C-limited conditions. This matches field conditions for the study system in which OC concentrations are low and porewater within saturated sediments is consistently aerobic (Stegen et al., 2018b). These results together suggest that the field data is consistently interpretable due to C limitation and O_2_ excess. In support of this, recent experimental work has shown a dependency of aerobic respiration on OM thermodynamics when C is limited but has shown no effect of C thermodynamics on respiration when C is widely available (Garayburu-Caruso et al., 2020).

In conventional dynamic biogeochemical modeling approaches, the difficulty in finding reliable parameter values is often a major hurdle in scaling up to large-scale complex systems because the number of kinetic constants is increasing in proportion to the number of compounds. As mentioned earlier, this barrier is overcome by our modeling approach that formulates compound-specific reaction rates with only two parameters, i.e., maximal growth rate (μ^*max*^) [1/h] or [1/d] and harvest volume (*V*_*h*_) [m^3^] or [L]. They are tunable parameters, which are quantitatively determinable via data fitting, e.g., when dynamic concentration profiles of substrates are available. Our model can guide experimental design to identify parameters and understand factors driving their variation across systems. In the present study using OM profiles from FTICR-MS, lack of quantitative information (i.e., concentrations) on individual OM molecules is an intrinsic limitation. Consequently, we focused on evaluating carbon *quality* based on the normalized distribution of OM, but this gap can be filled for improving predictions, e.g., through the integration with other complementary metabolomics approaches that can provide quantitative data (Hertkorn et al., 2013; McCallister et al., 2018).

The capability of our method that incorporates high-resolution mass spectrometry (or OM characterization methods) data into biogeochemical modeling greatly facilitates other research programs in the field that collect OM chemistry datasets. The Worldwide Hydrobiogeochemistry Observation Network for Dynamic River Systems (WHONDRS), for example, is a global research consortium that aims at understanding cross-scale dynamic interactions among hydrology, biogeochemistry, and microbiology in river corridors (Stegen and Goldman, 2018). As an initial effort, WHONDRS provides the collection of high-resolution OM profiles such as FTICR-MS data across rivers in the world (Stegen et al., 2018a; Chu et al., 2019; Danczak et al., 2019, 2020; Garayburu-Caruso et al., 2019; Goldman et al., 2019; Renteria et al., 2019; Stegen et al., 2019; Wells et al., 2019). Other networked efforts, such as the National Ecological Observation Network (Teeri and Raven, 2002; Barnett et al., 2019), provides similar kinds of data that are amenable for analysis via SXM. Analysis of these data, which are collected and analyzed consistently across systems, using the SXM framework will significantly improve our understanding of the level of heterogeneity across space and time in OM consumption and respiration, and thus could be used as a critical tool for more mechanistic predictions of spatial and temporal variation in stream/river CO_2_ emissions and other coupled biogeochemical rates from local to global scales.

**FIGURE 10:**
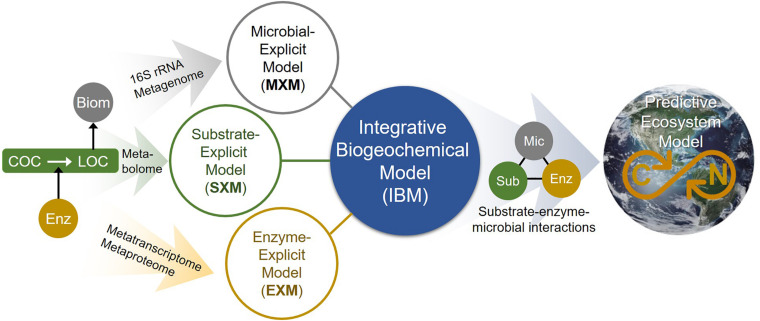
The integrated biogeochemical modeling (IBM) concept that combines MXM, SXM, and EXM, which are respective representations of significant expansions from typical lumped models by integrating multi-omics data to identify functional contributions of individual organisms and/or functional guilds, substrate-specific degradation pathways, and detailed enzymatic processes. Consequently, the IBM may enable providing a mechanistic understanding of dynamic linkage and interactions among substrates, enzymes, and microbes at a molecular level and significantly improving the performance of complex ecosystem models in predicting OC consumption and CO_2_ emission in space and time. COC, complex organic carbon; LOC, labile organic carbon; Enz, enzyme; Biom, biomass; Mic, microbes; and Sub, substrates.

As shown, accounting for enzymatic regulation in SXMs is critical for more reasonable simulations. Considering metabolic regulation can be best described by EXMs with direct association with specific enzymes, this results suggests that a synergistic integration of SXMs with other existing frameworks is an important future direction to fill the gaps that each approach has and to address advanced science questions that could not be addressed individually. We illustrate a conceptual master platform of integrative biogeochemical modeling (IBM) that accounts for the interaction among substrates, microbes, and enzymes at an unprecedented level of detail (Figure 10). We envision that the IBM will significantly contribute to create a molecular-level understanding of biogeochemistry that can be translated to complex ecosystem modeling. In this regard, the SXM concept proposed in this work provides a key component of IBM that has been missing so far.

## Data Availability Statement

All relevant data and numerical codes including KBase apps for computing thermodynamic parameters and reaction rates are available at: https://github.com/hyunseobsong/lambda.

## Author Contributions

H-SS, JCS, EBG, VAG-C, and TDS conceptualized the study. H-SS developed the codes and performed the theoretical analyses. J-YL, XC, and JDM developed the SDK Apps for the implementation of the codes in KBase. J-YL and WCN contributed to the data processing. H-SS drafted out the manuscript, which was edited by JCS and EBG. All the authors contributed to the writing.

## Conflict of Interest

The authors declare that the research was conducted in the absence of any commercial or financial relationships that could be construed as a potential conflict of interest.

## References

[B1] Allison S. D. (2012). A trait-based approach for modelling microbial litter decomposition. *Ecol. Lett.* 15 1058–1070. 10.1111/j.1461-0248.2012.01807.x 22642621

[B2] Allison S. D. (2017). Building predictive models for diverse microbial communities in soil. *Microb. Biomass* 2017 141–166. 10.1142/9781786341310_0006

[B3] Amend J. P. LaRowe D. E. (2019). Minireview: demystifying microbial reaction energetics. *Environ. Microbiol.* 21 3539–3547. 10.1111/1462-2920.14778 31403238 PMC6852080

[B4] Barnett D. T. Duffy P. A. Schimel D. S. Krauss R. E. Irvine K. M. Davis F. W. (2019). The terrestrial organism and biogeochemistry spatial sampling design for the national ecological observatory network. *Ecosphere* 10:e02540 10.1002/ecs2.2540

[B5] Blankinship J. C. Berhe A. A. Crow S. E. Druhan J. L. Heckman K. A. Keiluweit M. (2018). Improving understanding of soil organic matter dynamics by triangulating theories, measurements, and models. *Biogeochemistry* 140 1–13. 10.1007/s10533-018-0478-2

[B6] Bouskill N. J. Tang J. Riley W. J. Brodie E. L. (2012). Trait-based representation of biological nitr fication: model development testing, and predicted community composition. *Front. Microbiol.* 3:364. 10.3389/fmicb.2012.00364 23087681 PMC3475126

[B7] Chu R. K. Goldman A. E. Brooks S. C. Danczak R. E. Garayburu-Caruso V. A. Graham E. B. (2019). *WHONDRS 48 Hour Diel Cycling Study at the East Fork Poplar Creek in Tennessee, USA. Environmental System Science Data Infrastructure for a Virtual Ecosystem [WHONDRS].* Berkeley, CA: ESS-DIVE.

[B8] Danczak R. E. Goldman A. E. Chu R. K. Garayburu-Caruso V. A. Graham E. B. He X. (2019). *WHONDRS 48 Hour Diel Cycling Study at the Altamaha River in Georgia, USA. Environmental System Science Data Infrastructure for a Virtual Ecosystem [WHONDRS].* Berkeley, CA: ESS-DIVE.

[B9] Danczak R. E. Goldman A. E. Chu R. K. Toyoda J. G. Garayburu-Caruso V. A. Tolic N. (2020). Deterministic processes drive spatiotemporal variation in stream corridor metabolites despite conserved chemical attributes. *bioRxiv* [Preprint], 10.1101/2020.02.12.946459v1.full

[B10] Desmond-Le Quemener E. Bouchez T. (2014). A thermodynamic theory of microbial growth. *ISME J.* 8 1747–1751. 10.1038/ismej.2014.7 24522260 PMC4817618

[B11] Dick J. M. (2019). CHNOSZ: thermodynamic calculations and diagrams for geochemistry. *Front. Earth Sci.* 7:180. 10.3389/fmicb.2012.00180 22783233 PMC3390585

[B12] Fatichi S. Manzoni S. Or D. Paschalis A. (2019). A mechanistic model of microbially mediated soil biogeochemical processes: a reality check. *Glob. Biogeochem. Cycles* 33 620–648. 10.1029/2018gb006077

[B13] Garayburu-Caruso V. Stegen J. Song H.-S. Renteria L. Wells J. Garcia W. (2020). Carbon limitation leads to thermodynamic regulation of aerobic metabolism. *Environ. Sci. Technol. Lett.* 7 517–524. 10.1021/acs.estlett.0c00258

[B14] Garayburu-Caruso V. A. Goldman A. E. Chu R. K. Danczak R. E. Graham E. B. Lin X. (2019). *WHONDRS 48 Hour Diel Cycling Study at the Columbia River in Washington, USA. Worldwide Hydrobiogeochemistry Observation Network for Dynamic River Systems (WHONDRS).* Berkeley, CA: ESS-DIVE.

[B15] Goldman A. E. Arnon S. Bar-Zeev E. Chu R. K. Danczak R. E. Garayburu-Caruso V. A. (2019). *WHONDRS 48 Hour Diel Cycling Study at the Jordan River, Israel. Environmental System Science Data Infrastructure for a Virtual Ecosystem.* Berkeley, CA: ESS-DIVE.

[B16] Gonzalez-Pinzon R. Haggerty R. Myrold D. D. (2012). Measuring aerobic respiration in stream ecosystems using the resazurin-resorufin system. *J. Geophys. Res. Biogeosci.* 117:965.

[B17] Graham E. B. Crump A. R. Kennedy D. W. Arntzen E. Fansler S. Purvine S. O. (2018). Multi ’omics comparison reveals metabolome biochemistry, not microbiome composition or gene expression, corresponds to elevated biogeochemical function in the hyporheic zone. *Sci. Total Environ.* 642 742–753. 10.1016/j.scitotenv.2018.05.256 29920461

[B18] Graham E. B. Crump A. R. Resch C. T. Fansler S. Arntzen E. Kennedy D. W. (2016). Coupling spatiotemporal community assembly processes to changes in microbial metabolism. *Front Microbiol.* 7:1949. 10.3389/fmicb.2016.01949 28123379 PMC5226446

[B19] Graham E. B. Goldman A. E. Crump A. R. Goldman A. E. Bramer L. M. Arntzen E. (2017). Carbon inputs from riparian vegetation limit oxidation of physically bound organic carbon via biochemical and thermodynamic processes. *J. Geophys. Res. Biogeosci.* 122 3188–3205. 10.1002/2017jg003967

[B20] Heijnen J. J. Vandijken J. P. (1993). In search of a thermodynamic description of biomass yields for the chemotropic growth of microorganisms - response. *Biotechnol. Bioeng.* 42 1127–1130. 10.1002/bit.260420916 18601018

[B21] Heijnen J. J. Vanloosdrecht M. C. M. Tijhuis L. (1992). A black-box mathematical-model to calculate autotrophic and heterotrophic biomass yields based on gibbs energy-dissipation. *Biotechnol. Bioeng.* 40 1139–1154. 10.1002/bit.260401003 18601065

[B22] Hertkorn N. Harir M. Koch B. P. Michalke B. Schmitt-Kopplin P. (2013). High-field NMR spectroscopy and FTICR mass spectrometry: powerful discovery tools for the molecular level characterization of marine dissolved organic matter. *Biogeosciences* 10 1583–1624. 10.5194/bg-10-1583-2013

[B23] Hood R. R. Laws E. A. Armstrong R. A. Bates N. R. Brown C. W. Carlson C. A. (2006). Pelagic functional group modeling: progress, challenges and prospects. *Deep Sea Res. Part Top. Stud. Oceanogr.* 53 459–512.

[B24] Jin Q. S. Roden E. E. (2011). Microbial physiology-based model of ethanol metabolism in subsurface sediments. *J. Contamin. Hydrol.* 125 1–12. 10.1016/j.jconhyd.2011.04.002 21652106

[B25] Keenleyside W. (2019). *Microbiology: Canadian Edition.* New York, NY: Simple Book Publishing.

[B26] Kleerebezem R. Van Loosdrecht M. C. M. (2010). A generalized method for thermodynamic state analysis of environmental systems. *Crit. Rev. Environ. Sci. Technol.* 40 1–54. 10.1080/10643380802000974

[B27] Kujawinski E. B. Behn M. D. (2006). Automated analysis of electrospray ionization Fourier transform ion cyclotron resonance mass spectra of natural organic matter. *Analyt. Chem.* 78 4363–4373. 10.1021/ac0600306 16808443

[B28] Larowe D. E. Amend J. P. (2015). Catabolic rates, population sizes and doubling/replacement times of microorganisms in natural settings. *Am. J. Sci.* 315 167–203. 10.2475/03.2015.01

[B29] LaRowe D. E. Amend J. P. (2016). The energetics of anabolism in natural settings. *ISME J.* 10 1285–1295. 10.1038/ismej.2015.227 26859771 PMC5029197

[B30] LaRowe D. E. Van Cappellen P. (2011). Degradation of natural organic matter: a thermodynamic analysis. *Geochim. Cosmochim. Acta* 75 2030–2042. 10.1016/j.gca.2011.01.020

[B31] Manzoni S. Moyano F. Katterer T. Schimel J. (2016). Modeling coupled enzymatic and solute transport controls on decomposition in drying soils. *Soil Biol. Biochem.* 95 275–287. 10.1016/j.soilbio.2016.01.006

[B32] McCallister S. Ishikawa N. Kothawala D. (2018). Biogeochemical tools for characterizing organic carbon in inland aquatic ecosystems. *Limnol. Oceanogr. Lett.* 3 444–457. 10.1002/lol2.10097

[B33] McCarty P. L. (2007). Thermodynamic electron equivalents model for bacterial yield prediction: modifications and comparative evaluations. *Biotechnol. Bioeng.* 97 377–388. 10.1002/bit.21250 17089390

[B34] Minor E. C. Steinbring C. J. Longnecker K. Kujawinski E. B. (2012). Characterization of dissolved organic matter in Lake superior and its watershed using ultrahigh resolution mass spectrometry. *Organ. Geochem.* 43 1–11. 10.1016/j.orggeochem.2011.11.007

[B35] Moorhead D. L. Rinkes Z. L. Sinsabaugh R. L. Weintraub M. N. (2013). Dynamic relationships between microbial biomass, respiration, inorganic nutrients and enzyme activities: informing enzyme-based decomposition models. *Front. Microbiol.* 4:223. 10.3389/fmicb.2012.00223 23964272 PMC3740267

[B36] Paul E. A. (2016). The nature and dynamics of soil organic matter: plant inputs, microbial transformations, and organic matter stabilization. *Soil Biol. Biochem.* 98 109–126. 10.1016/j.soilbio.2016.04.001

[B37] Ramkrishna D. Song H.-S. (2012). Dynamic models of metabolism: review of the cybernetic approach. *Aiche. J.* 58 986–997. 10.1002/aic.13734

[B38] Ramkrishna D. Song H.-S. (2018). *Cybernetic Modeling for Bioreaction Engineering.* Cambridge: Cambridge University Press.

[B39] Renteria L. Goldman A. E. Chu R. K. Danczak R. E. Garayburu-Caruso V. A. Graham E. B. (2019). *WHONDRS 48 Hour Diel Cycling Study at the Nisqually River, WA. Environmental System Science Data Infrastructure for a Virtual Ecosystem.* Berkeley, CA: ESS-DIVE.

[B40] Rittmann B. E. McCarty P. L. (2012). *Environmental Biotechnology: Principles and Applications.* New York, NY: Tata McGraw-Hill Education.

[B41] Schimel J. P. Schaeffer S. M. (2012). Microbial control over carbon cycling in soil. *Front. Microbiol.* 3:348. 10.3389/fmicb.2012.00348 23055998 PMC3458434

[B42] Slater L. D. Ntarlagiannis D. Day-Lewis F. D. Mwakanyamale K. Versteeg R. J. Ward A. (2010). Use of electrical imaging and distributed temperature sensing methods to characterize surface water-groundwater exchange regulating uranium transport at the Hanford 300 Area, Washington. *Water Resour. Res.* 46:W10533 10.1029/2010WR009110

[B43] Song H.-S. Cannon W. Beliaev A. Konopka A. (2014). Mathematical modeling of microbial community dynamics: a methodological review. *Processes* 2 711–752. 10.3390/pr2040711

[B44] Song H.-S. Liu C. X. (2015). Dynamic metabolic modeling of denitrifying bacterial growth: the cybernetic approach. *Indust. Eng. Chem. Res.* 54 10221–10227. 10.1021/acs.iecr.5b01615

[B45] Song H.-S. Ramkrishna D. (2010). Prediction of metabolic function from limited data: lumped hybrid cybernetic modeling (L-HCM). *Biotechnol. Bioeng.* 106 271–284. 20148411 10.1002/bit.22692

[B46] Song H.-S. Ramkrishna D. (2011). Cybernetic models based on lumped elementary modes accurately predict strain-specific metabolic function. *Biotechnol. Bioeng.* 108 127–140. 10.1002/bit.22922 20830732

[B47] Song H.-S. Stegen J. C. Graham E. B. Lee J.-Y. Garayburu-Caruso V. A. Nelson W. C. (2020). Representing organic matter thermodynamics in biogeochemical reactions via substrate-explicit modeling. *bioRxiv* [Preprint], 10.1101/2020.02.27.968669v1 PMC7644784 33193121

[B48] Song H.-S. Thomas D. G. Stegen J. C. Li M. J. Liu C. X. Song X. H. (2017). Regulation-structured dynamic metabolic model provides a potential mechanism for delayed enzyme response in denitrification process. *Front. Microbiol.* 8:1866 10.3389/fmicb.2012.01866 PMC5627231 29046664

[B49] Song X. H. Chen X. Y. Stegen J. Hammond G. Song H.-S. Dai H. (2018). Drought Conditions maximize the impact of high-frequency flow variations on thermal regimes and biogeochemical function in the Hyporheic zone. *Water Resour. Res.* 54 7361–7382. 10.1029/2018wr022586

[B50] Stegen J. C. Goldman A. E. (2018). WHONDRS: a community resource for studying dynamic river corridors. *mSystems* 3:e0151-18. 10.1128/mSystems.00151-18 PMC6178584 30320221

[B51] Stegen J. C. Goldman A. E. Blackburn S. E. Chu R. K. Danczak R. E. Garayburu-Caruso V. A. (2018a). *WHONDRS Surface Water Sampling for Metabolite Biogeography. Environmental System Science Data Infrastructure for a Virtual Ecosystem.* Berkeley, CA: ESS-DIVE.

[B52] Stegen J. C. Johnson T. Fredrickson J. K. Wilkins M. J. Konopka A. E. Nelson W. C. (2018b). Influences of organic carbon speciation on hyporheic corridor biogeochemistry and microbial ecology. *Nat. Commun.* 9:1034. 10.1038/s41467-018-03572-7 PMC5841274 29515121

[B53] Stegen J. C. Goldman A. E. Chu R. K. Danczak R. E. Garayburu-Caruso V. A. Graham E. B. (2019). *WHONDRS 48 Hour Diel Cycling Study at HJ Andrews Experimental Forest Watershed 1 (WS1). Environmental System Science Data Infrastructure for a Virtual Ecosystem.* Berkeley, CA: ESS-DIVE.

[B54] Stephanopoulos G. Aristidou A. A. Nielsen J. (1998). *Metabolic Engineering: Principles and Methodologies.* Amsterdam: Elsevier.

[B55] Sulman B. N. Moore J. A. M. Abramoff R. Averill C. Kivlin S. Georgiou K. (2018). Multiple models and experiments underscore large uncertainty in soil carbon dynamics. *Biogeochemistry* 141 109–123. 10.1007/s10533-018-0509-z

[B56] Sulman B. N. Phillips R. P. Oishi A. C. Shevliakova E. Pacala S. W. (2014). Microbe-driven turnover offsets mineral-mediated storage of soil carbon under elevated CO2. *Nat. Clim. Chang.* 4 1099–1102. 10.1038/nclimate2436

[B57] Tang J. Y. Riley W. J. (2015). Weaker soil carbon-climate feedbacks resulting from microbial and abiotic interactions. *Nat. Clim. Chang.* 5 56–60. 10.1038/nclimate2438

[B58] Teeri J. A. Raven P. H. (2002). A national ecological observatory network. *Science* 298 1893–1893. 10.1126/science.298.5600.1893 12474846

[B59] Tfaily M. M. Chu R. K. Toyoda J. Tolic N. Robinson E. W. Pasa-Tolic L. (2017). Sequential extraction protocol for organic matter from soils and sediments using high resolution mass spectrometry. *Analyt. Chim. Acta* 972 54–61. 10.1016/j.aca.2017.03.031 28495096

[B60] Todd-Brown K. E. O. Hopkins F. M. Kivlin S. N. Talbot J. M. Allison S. D. (2012). A framework for representing microbial decomposition in coupled climate models. *Biogeochemistry* 109 19–33. 10.1007/s10533-011-9635-6

[B61] Varjani S. J. (2017). Microbial degradation of petroleum hydrocarbons. *Bioresou. Technol.* 223 277–286. 10.1016/j.biortech.2016.10.037 27789112

[B62] Wang G. S. Jagadamma S. Mayes M. A. Schadt C. W. Steinweg J. M. Gu L. H. (2015). Microbial dormancy improves development and experimental validation of ecosystem model. *ISME J.* 9 226–237. 10.1038/ismej.2014.120 25012899 PMC4274429

[B63] Wells J. R. Goldman A. E. Chu R. K. Danczak R. E. Garayburu-Caruso V. A. Graham E. B. (2019). *WHONDRS 48 Hour Diel Cycling Study at the Erpe River, Germany. Environmental System Science Data Infrastructure for a Virtual Ecosystem.* Berkeley, CA: ESS-DIVE.

[B64] Wieder W. R. Allison S. D. Davidson E. A. Georgiou K. Hararuk O. He Y. J. (2015). Explicitly representing soil microbial processes in Earth system models. *Glob. Biogeochem. Cycles* 29 1782–1800. 10.1002/2015gb005188

[B65] Wieder W. R. Grandy A. S. Kallenbach C. M. Bonan G. B. (2014). Integrating microbial physiology and physio-chemical principles in soils with the MIcrobial-MIneral carbon stabilization (MIMICS) model. *Biogeosciences* 11 3899–3917. 10.5194/bg-11-3899-2014

[B66] Zachara J. M. Long P. E. Bargar J. Davis J. A. Fox P. Fredrickson J. K. (2013). Persistence of uranium groundwater plumes: contrasting mechanisms at two DOE sites in the groundwater–river interaction zone. *J. Contam. Hydrol.* 147, 45–72. 10.1016/j.jconhyd.2013.02.001 23500840

