# Supplementary material for: Representing Organic Matter Thermodynamics in Biogeochemical Reactions via Substrate-Explicit Modeling
Source: Front Microbiol. 2020 Oct 23;11:531756. doi: 10.3389/fmicb.2020.531756 (PMC7644784; doi:10.3389/fmicb.2020.531756)
Supplement: Supplementary file 1 [file Data_Sheet_1.docx]

**Supplementary Information**

**Representing Organic Matter Thermodynamics in Biogeochemical Reactions via Substrate-Explicit Modeling**

Hyun-Seob Song^1,2*^, James C. Stegen^1^, Emily B. Graham^1^, Joon-Yong Lee^1^, Vanessa A. Garayburu-Caruso^1^, William C. Nelson^1^, Xingyuan Chen^1^, J. David Moulton^3^, Timothy D. Scheibe^1^

^1^Pacific Northwest National Laboratory, Richland, WA 99352

^2^University of Nebraska-Lincoln, Lincoln NE 68583

^3^Los Alamos National Laboratory, Los Alamos, NM 87545

^*^Corresponding author: hsong5@unl.edu; 402-472-1036

**Contents**

Supplementary text

- Derivation of catabolic reactions
- Derivation of anabolic reactions
- Derivation of reaction kinetics

Supplementary figures

- Supplementary Figure S1: Replot of Figure 2 with recalculation of thermodynamic functions per a unit C-mole of OC.
- Supplementary Figure S2: Choice of two representative sections for the comparison of the low- and high-activity zones based on the correlations of aerobic respiration with (a) the energy coupling parameter ($\lambda$) and O_2_ consumption rate ($|r_{O_{2}}|$).
- Supplementary Figure S3: Dynamic simulations based on 500 OC randomly selected respectively from the low activity (LA) and high activity (HA) nutrient pools.

**Supplementary Text**

**Derivation of catabolic reactions**

Catabolic reaction is obtained by combining Ed (i.e., OC) and Ea (i.e., O_2_) half reactions (denoted by $R^{D}$and $R^{A}$). Following (LaRowe and Van Cappellen, 2011), we write $R^{D}$as follows:

$$\begin{matrix} \text{R}^{\text{D}}: y_{OC}^{D}\text{OC}+y_{H_{2}O}^{D}\text{H}_{2}\text{O}+y_{HCO_{3}^{-}}^{D}\text{HCO}_{3}^{\text{-}}+y_{NH_{4}^{+}}^{D}\text{NH}_{4}^{\text{+}} \\ +y_{HPO_{4}^{2-}}^{D}\text{HPO}_{4}^{\text{2-}}+y_{HS^{-}}^{D}\text{HS}^{\text{-}}+y_{H^{+}}^{D}\text{H}^{\text{+}}+y_{e^{-}}^{D}\text{e}^{\text{-}}=0 \end{matrix}$$

The chemical formula for OC is represented as follows:

$$\text{OC}=\text{C}_{a}\text{H}_{b}\text{N}_{c}\text{O}_{d}\text{P}_{e}\text{S}_{f}^{z}$$

where the subscripts *a, b, c, d, e*, and *f* denote the elemental composition of OC in terms of C, H, N, O, P, and S, and the superscript *z* represents the net charge of OC. Stoichiometric coefficients in Eq. are functions of elemental composition of OC (i.e., *a, b, c, d, e*, *f*, and *z*) (LaRowe and Van Cappellen, 2011). The Ea half reaction $\text{R}^{\text{A}}$ is given in a simple form as follows (Rittmann and McCarty, 2012):

$$\text{R}^{\text{A}}:-\text{O}_{2}-4\text{H}^{\text{+}}-4\text{e}^{\text{-}}+2\text{H}_{2}\text{O}=0$$

These reactions can be represented in terms of the following two vectors of stoichiometric coefficients, i.e.,

$$\mathbf{y}^{\mathbf{D}}\equiv\left[\begin{matrix} y_{OC}^{D} \\ y_{H_{2}O}^{D} \\ y_{HCO_{3}^{-}}^{D} \\ y_{NH_{4}^{+}}^{D} \\ y_{HPO_{4}^{2-}}^{D} \\ y_{HS^{-}}^{D} \\ y_{H^{+}}^{D} \\ y_{e^{-}}^{D} \\ y_{O_{2}}^{D} \\ y_{Biom}^{D} \end{matrix}\right]=\left[\begin{matrix} -1 \\ -\left(3a+4e-d\right) \\ a \\ c \\ e \\ f \\ \left(5a+b-4c-2d+7e-f\right) \\ \left(-z+4a+b-3c-2d+5e-2f\right) \\ 0 \\ 0 \end{matrix}\right], \mathbf{y}^{\mathbf{A}}\equiv\left[\begin{matrix} y_{OC}^{A} \\ y_{H_{2}O}^{A} \\ y_{HCO_{3}^{-}}^{A} \\ y_{NH_{4}^{+}}^{A} \\ y_{HPO_{4}^{2-}}^{A} \\ y_{HS^{-}}^{A} \\ y_{H^{+}}^{A} \\ y_{e^{-}}^{A} \\ y_{O_{2}}^{A} \\ y_{Biom}^{A} \end{matrix}\right]=\left[\begin{matrix} 0 \\ 2 \\ 0 \\ 0 \\ 0 \\ 0 \\ -4 \\ -4 \\ -1 \\ 0 \end{matrix}\right]$$

The overall stoichiometry of the complete catabolic reaction equation ($R^{Cat}$) is obtained by combining the Ed and Ea half reactions:

$$\text{R}^{\text{Cat}}=\sum_{i=1}^{n} y_{S_{i}}^{Cat}\text{S}_{\text{i}}$$

where the stoichiometric coefficient vector is determined as follows such that the net electron production or consumption through $R^{Cat}$ is zero, i.e.,

$$\mathbf{y}^{\mathbf{Cat}}=\frac{1}{y_{e^{-}}^{D}y_{OC}^{A}-y_{e^{-}}^{A}y_{OC}^{D}}\left(y_{e^{-}}^{A}\mathbf{y}^{\mathbf{D}}-y_{e^{-}}^{D}\mathbf{y}^{\mathbf{A}}\right)$$

**Derivation of anabolic reactions**

In anabolism, the substrates (carbon source and nitrogen source) are reduced to biomass. When the carbon source is the same as the electron donor (i.e., OC) (which is true for heterotrophic organisms) and the nitrogen source is ammonium, the anabolic reaction equation ($\text{R}^{\text{An*}}$) can be written as follows:

$$\begin{matrix} \text{R}^{\text{An*}}: y_{OC}^{An*}\text{OC}+y_{H_{2}O}^{An*}\text{H}_{2}\text{O}+y_{HCO_{3}^{-}}^{An*}\text{HCO}_{3}^{\text{-}}+y_{NH_{4}^{+}}^{An*}\text{NH}_{4}^{\text{+}} \\ +y_{HPO_{4}^{2-}}^{An*}\text{HPO}_{4}^{\text{2-}}+y_{HS^{-}}^{An*}\text{HS}^{\text{-}}+y_{H^{+}}^{An*}\text{H}^{\text{+}}+y_{e^{-}}^{An*}\text{e}^{\text{-}}+\text{Biom}=0 \end{matrix}$$

Similar to previous cases, the vector of stoichiometric coefficients in $\text{R}^{\text{An*}}$ (denoted by $\mathbf{y}^{\mathbf{An*}}$) can also be represented as a function of composition of OC to meet elemental mass balances, i.e.,

$$\mathbf{y}^{\mathbf{An*}}\equiv\left[\begin{matrix} y_{OC}^{An*} \\ y_{H_{2}O}^{An*} \\ y_{HCO_{3}^{-}}^{An*} \\ y_{NH_{4}^{+}}^{An*} \\ y_{HPO_{4}^{2-}}^{An*} \\ y_{HS^{-}}^{An*} \\ y_{H^{+}}^{An*} \\ y_{e^{-}}^{An*} \\ y_{O_{2}}^{An*} \\ y_{Biom}^{An*} \end{matrix}\right]=\left[\begin{matrix} -1/a \\ -(a-2d+8e)/(2a) \\ 0 \\ c/a-1/5 \\ e/a \\ f/a \\ -(4c-b+2d-7e+f)/a \\ -(a-5b+15c+10d-25e+10f+5z)/(5a) \\ 0 \\ 1 \end{matrix}\right]$$

Note that $y_{e^{-}}^{An*}$can be zero, negative or positive depending on the difference of oxidation states between the biomass and substrates (denoted by $\gamma_{Biom}$ and $\gamma_{Sub}$). If $\gamma_{Biom}<\gamma_{Sub}$ (or $y_{e^{-}}^{An*}<0$), it implies electrons are required to convert substrates to biomass; if $\gamma_{Biom}>\gamma_{Sub}$ (or $y_{e^{-}}^{An*}>0$), the conversion of substrates to biomass produces electrons, requiring an electron acceptor. In combination with electron donor or acceptor equation defined in Eqs. , , and , the anabolic reaction equation ($R^{An}$) is finally represented without the electron term as follows:

$$\begin{matrix} \text{R}^{\text{An}}: y_{OC}^{An}\text{OC}+y_{H_{2}O}^{An}\text{H}_{2}\text{O}+y_{HCO_{3}^{-}}^{An}\text{HCO}_{3}^{\text{-}}+y_{NH_{4}^{+}}^{An}\text{NH}_{4}^{\text{+}} \\ +y_{HPO_{4}^{2-}}^{An}\text{HPO}_{4}^{\text{2-}}+y_{HS^{-}}^{An}\text{HS}^{\text{-}}+y_{H^{+}}^{An}\text{H}^{\text{+}}+\text{Biom}=0 \end{matrix}$$

where the vector of stoichiometric coefficients is given as follows:

$$\mathbf{y}^{\mathbf{An}}=\left\{\begin{matrix} \mathbf{y}^{\mathbf{An*}}-\left(\text{y}_{e^{-}}^{An*}/y_{e^{-}}^{A}\right)\mathbf{y}^{\mathbf{A}}, & \text{if y}_{e^{-}}^{An*}>0 \\ \mathbf{y}^{\mathbf{An*}}-\left(\text{y}_{e^{-}}^{An*}/y_{e^{-}}^{D}\right)\mathbf{y}^{\mathbf{D}}, & \text{if y}_{e^{-}}^{An*}<0 \\ \mathbf{y}^{\mathbf{An*}}, & \text{if y}_{e^{-}}^{An*}=0 \end{matrix}\right.$$

**Reaction kinetics**

The foregoing sections show how to systematically convert chemical formula of OC to stoichiometric equations for oxidative OC degradation reactions including catabolic, anabolic, and metabolic reactions. As a next step, we extend it to formulate kinetics using the microbial growth thermodynamic theory developed by Desmond-Le Quemener and Bouchez (2014). They considered microbial growth through the following two steps: (1) reversible transition of a microbe ($\text{X}$) to an activated state ($\text{X}^{\text{‡}}$) and (2) irreversible cell division from the activated cell to two daughter cells, i.e.,

$$\text{X}\leftrightarrow\text{X}^{\text{‡}}\to2\text{X}$$

Similar to the classical transition state theory in chemical reaction (Truhlar et al., 1996), the reversible reaction in the first step is assumed to be faster than the second step so that they are in equilibrium. During the first step, each microbe harvests chemical energy from environment. In order to trigger cell division, the energy acquisition from environment should exceed a certain threshold level ($E^{\ddagger}$, activation energy). The activation energy is nothing but the summation of anabolic energy requirement and energy dissipation, i.e.,

$$E^{\ddagger}=\left(-\Delta G_{r,An}\right)+\left(-\Delta G_{r,Dis}\right)$$

The total usable energy for growth depends on two factors: (1) the energy generation through catabolism ($\Delta G_{r,Cat}$), and (2) the availability of energy sources (i.e., substrates) in environment. The second factor depends on substrate concentration ($[\text{S}]$) and the volume that a microbe can access for harvesting energy ($V_{h}$, harvest volume). Using statistical analysis, Desmond-Le Quemener and Bouchez showed that the equation for microbial growth rate ($\mu$) can be formulated as follows:

$$\mu=\mu^{\max}\exp\left(-\frac{(-\Delta G_{r,An})+(-\Delta G_{r,Dis})}{V_{h}[\text{S}]\Delta G_{r,Cat}}\right)$$

where $\mu^{\max}$ is the maximal specific growth rate. While conventional representation of microbial state is based on the rate of energy acquisition from the environment (Bradley et al., 2018), the formulation above links the microbial growth rate to a function of activation energy. Eq. can be rewritten in terms of $\lambda$ defined in Eq. (4) in the main text, i.e.,

$$\mu=\mu^{\max}\exp\left(-\frac{\lambda}{V_{h}[\text{S}]}\right)$$

In our current formulation where all stoichiometric equations were derived for a unit C-mole of biomass, the negative value of the parameter $\lambda$ is equal to the stoichiometry coefficient of OC. For the $i^{th}$ OC (i.e., $\text{OC}_{i}$), this leads to the following form of microbial growth rate:

$$\mu_{i}=\mu^{\max}\exp\left(-\frac{\left|y_{OC,i}\right|}{V_{h}[\text{OC}_{i}]}\right)$$

where $\mu_{i}$ is the microbial growth rate on $OC_{i}$, $y_{OC,i}$ is the stoichiometric coefficient of $OC_{i}$ in the metabolic reaction, and $\left|y_{OC,i}\right|$ denotes the absolution value of $y_{OC,i}$. Desmond-Le Quemener and Bouchez also provided the extension of the formulation to multiple substrates. In the case of oxidative degration of OC, the final growth equation becomes

$$\mu_{i}=\mu^{\max}\exp\left(-\frac{\left|y_{OC,i}\right|}{V_{h}[\text{OC}_{i}]}\right)\exp\left(-\frac{\left|y_{O_{2},i}\right|}{V_{h}[\text{O}_{2}]}\right)$$

where $y_{O_{2},i}$ is the stoichiometric coefficient of O_2_ in the metabolic reaction associated with oxidative degradation of $OC_{i}$.

**References**

Bradley, J.A., Amend, J.P., and Larowe, D.E. (2018). Bioenergetic Controls on Microbial Ecophysiology in Marine Sediments. *Frontiers in Microbiology* 9.

Larowe, D.E., and Van Cappellen, P. (2011). Degradation of natural organic matter: A thermodynamic analysis. *Geochimica Et Cosmochimica Acta* 75**,** 2030-2042.

Rittmann, B.E., and Mccarty, P.L. (2012). *Environmental biotechnology: principles and applications.* Tata McGraw-Hill Education.

Truhlar, D.G., Garrett, B.C., and Klippenstein, S.J. (1996). Current status of transition-state theory. *Journal of Physical Chemistry* 100**,** 12771-12800.

**Supplementary Figures**

**
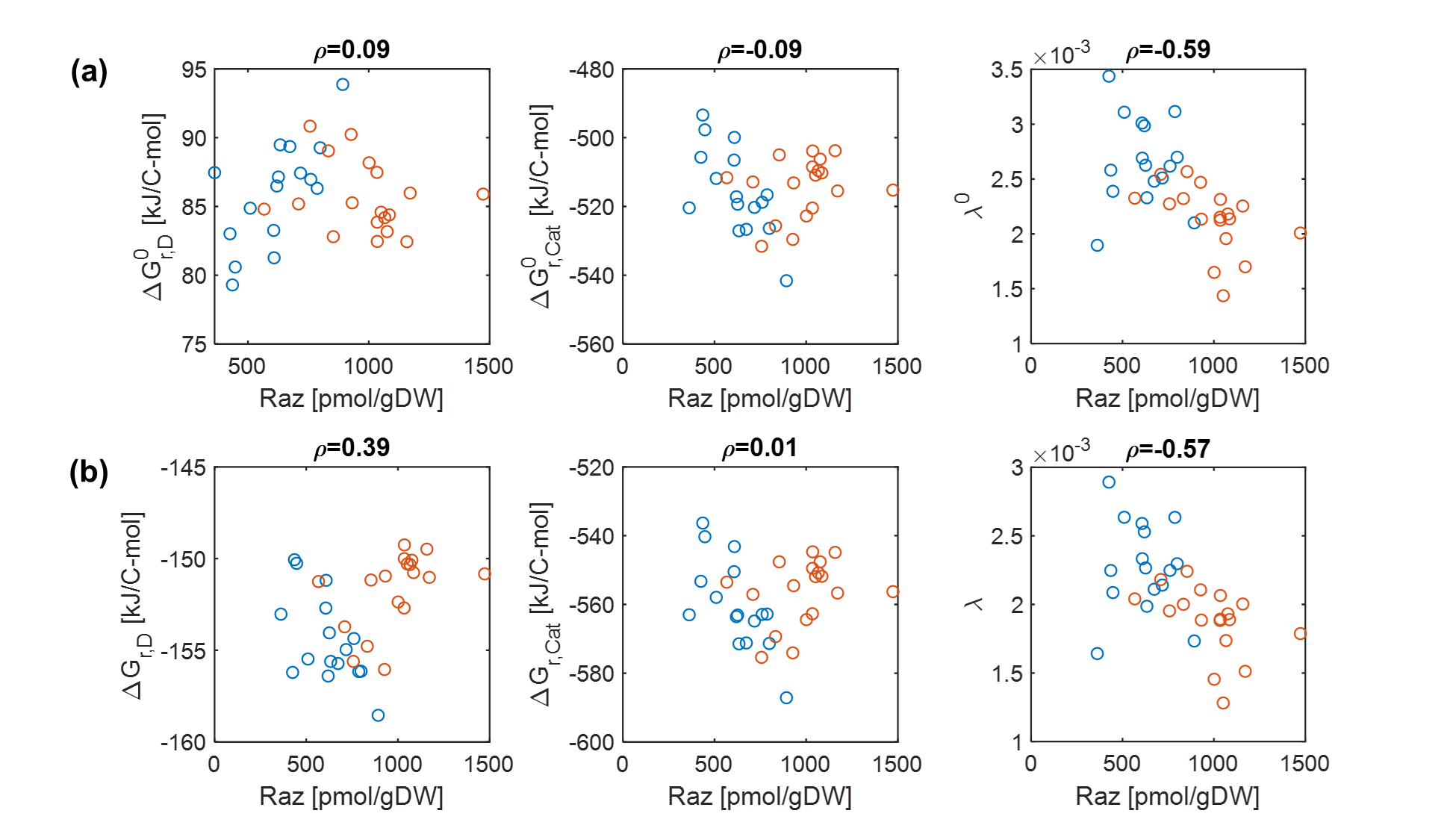
**

**Supplementary Figure S1.** Replot of Figure 2 with recalculation of thermodynamic functions per a unit C-mole of OC: (a) pH=0 (denoted by the superscript 0) and (b) pH=7. Blue and orange open circles denote the samples from low and high activity zones. Raz denotes the amount of resazurin reduced to resorufin over the 48hr incubation period.


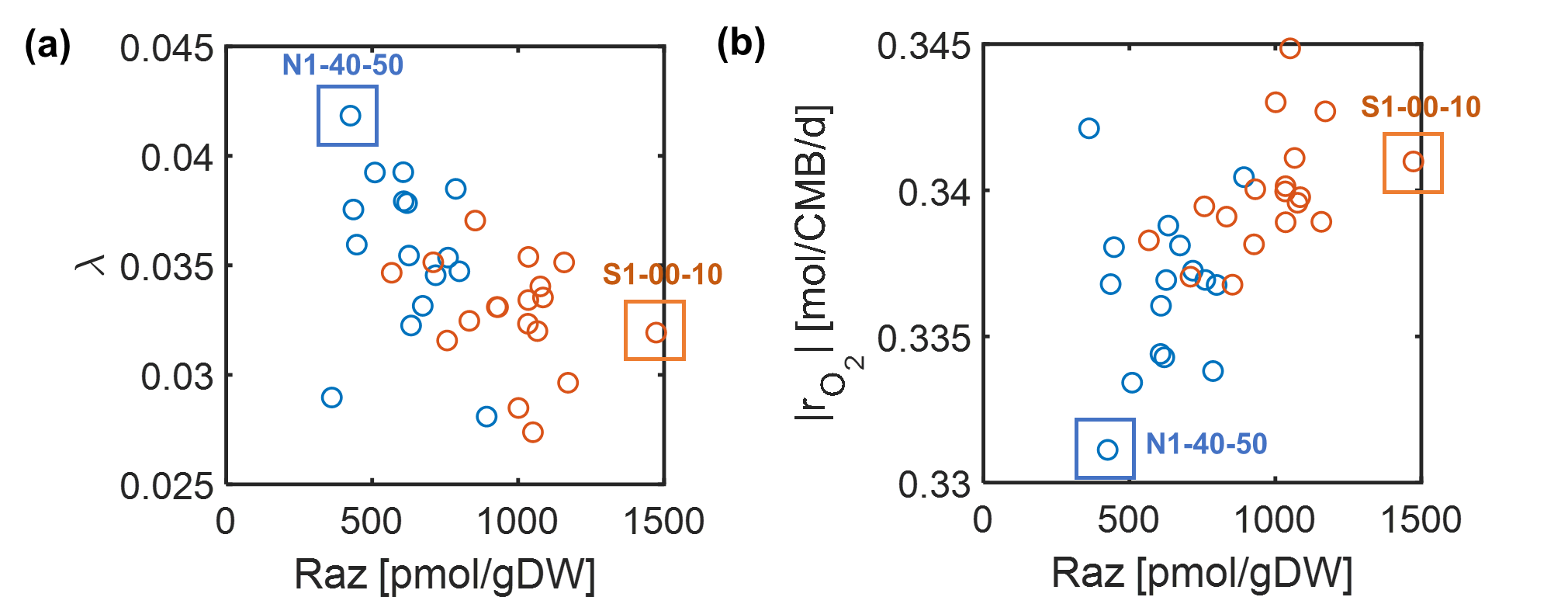


**Supplementary Figure S2.** Choice of two representative sections for the comparison of the low- and high-activity zones based on the correlations of aerobic respiration with (a) the energy coupling parameter ($\lambda$) and O_2_ consumption rate ($|r_{O_{2}}|$).Raz denotes the amount of resazurin reduced to resorufin over the 48hr incubation period. Blue and orange open circles denote the samples from low and high activity zones.


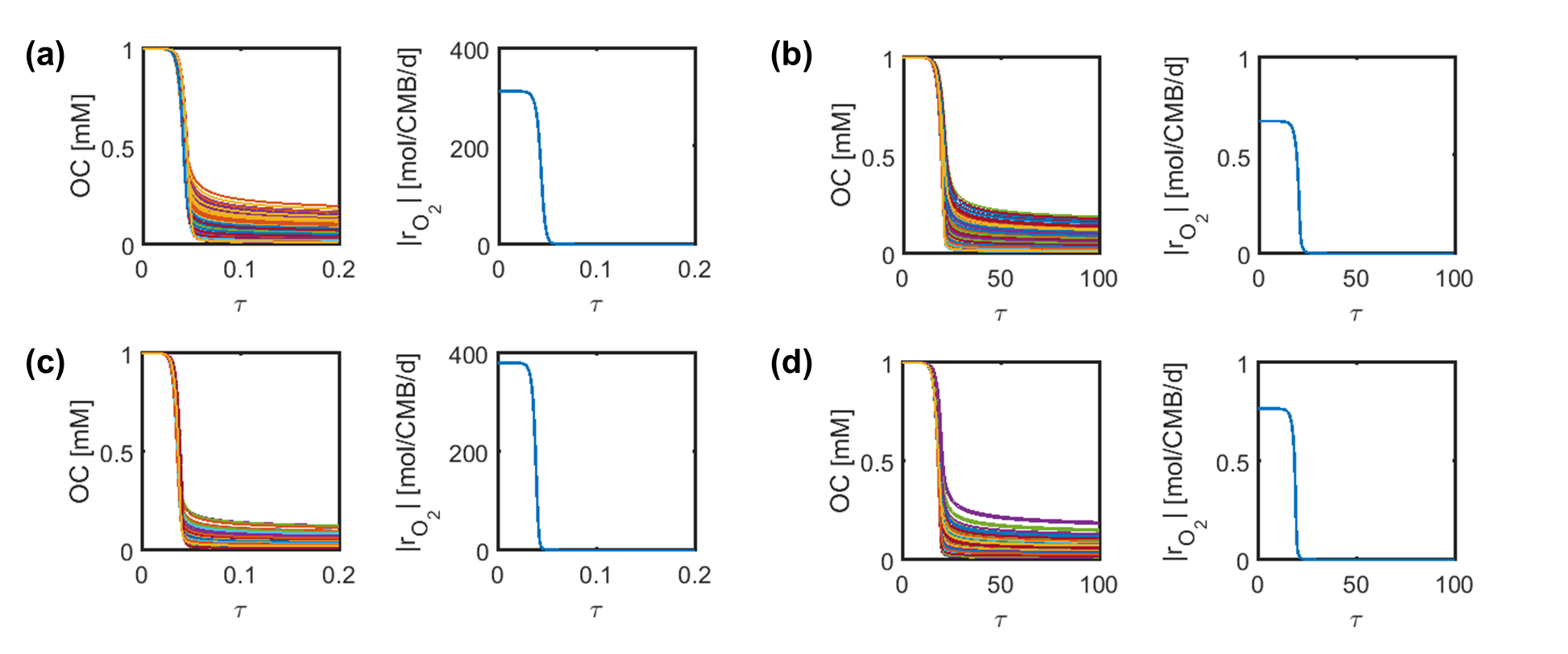


**Supplementary Figure S3.** Dynamic simulations based on 500 OC randomly selected respectively from the low activity (LA) and high activity (HA) nutrient pools: (a) and (b) LA sample: SXM (a) without and (b) with enzymatic regulation; (c) and (d) HA sample: SXM (c) without and (d) with enzymatic regulation. $\tau$ denotes the dimensionless time ($\equiv t\mu^{\max}$ where t =time and $\mu^{\max}$ = maximal growth rates). CMB = C-mole biomass.
